# The structural differences between patient-derived α-synuclein strains dictate characteristics of Parkinson’s disease, multiple system atrophy and dementia with Lewy bodies

**DOI:** 10.1007/s00401-020-02157-3

**Published:** 2020-04-30

**Authors:** Anke Van der Perren, Géraldine Gelders, Alexis Fenyi, Luc Bousset, Filipa Brito, Wouter Peelaerts, Chris Van den Haute, Steve Gentleman, Ronald Melki, Veerle Baekelandt

**Affiliations:** 1 Laboratory for Neurobiology and Gene Therapy, Department of Neurosciences, KU Leuven, Leuven, Belgium; 2 Institut François Jacob (MIRCen), CEA, and Laboratory of Neurodegenerative Diseases, CNRS, Fontenay-aux-Roses, France; 3 KU Leuven, Leuven Viral Vector Core, Leuven, Belgium; 4 Neuropathology Unit, Division of Brain Sciences, Department of Medicine, Imperial College London, London, UK

**Keywords:** Synucleinopathies, α-synuclein, Strains, Neurodegenerative disorders

## Abstract

Synucleinopathies, such as Parkinson’s disease (PD), multiple system atrophy (MSA), and dementia with Lewy bodies (DLB), are defined by the presence of α-synuclein (αSYN) aggregates throughout the nervous system but diverge from one another with regard to their clinical and pathological phenotype. The recent generation of pure fibrillar αSYN polymorphs with noticeable differences in structural and phenotypic traits has led to the hypothesis that different αSYN strains may be in part responsible for the heterogeneous nature of synucleinopathies. To further characterize distinct αSYN strains in the human brain, and establish a structure-pathology relationship, we pursued a detailed comparison of αSYN assemblies derived from well-stratified patients with distinct synucleinopathies. We exploited the capacity of αSYN aggregates found in the brain of patients suffering from PD, MSA or DLB to seed and template monomeric human αSYN in vitro via a protein misfolding cyclic amplification assay. A careful comparison of the properties of total brain homogenates and pure in vitro amplified αSYN fibrillar assemblies upon inoculation in cells and in the rat brain demonstrates that the intrinsic structure of αSYN fibrils dictates synucleinopathies characteristics. We report that MSA strains show several similarities with PD strains, but are significantly more potent in inducing motor deficits, nigrostriatal neurodegeneration, αSYN pathology, spreading, and inflammation, reflecting the aggressive nature of this disease. In contrast, DLB strains display no or only very modest neuropathological features under our experimental conditions. Collectively, our data demonstrate a specific signature for PD, MSA, and DLB-derived strains that differs from previously described recombinant strains, with MSA strains provoking the most aggressive phenotype and more similarities with PD compared to DLB strains.

## Introduction

Parkinson’s disease (PD), multiple system atrophy (MSA) and dementia with Lewy bodies (DLB) are neurodegenerative disorders which impose a considerable burden on our aging society. These three disorders, grouped together under the banner of synucleinopathies, are defined by the presence of α-synuclein (αSYN) aggregates throughout the nervous system but diverge from one another with regard to their clinical and pathological phenotype [19, 20]. PD is typically characterized by a long prodromal phase followed by parkinsonian motor features including bradykinesia, rigidity, and resting tremor [43]. During later stages, dementia affects more than half of all PD patients [1, 24]. The clinical manifestation of DLB is similar to PD but cognitive symptoms emerge typically one year before onset of motor symptoms [32]. Conversely, MSA is mainly characterized by autonomic dysfunction and cerebellar or parkinsonian features [18]. In PD and DLB, αSYN mainly forms large neuronal inclusions called Lewy bodies (LBs) and neurites (LNs) [51, 59], while in MSA, αSYN aggregates are mostly found in the oligodendroglia as glial cytoplasmic inclusions (GCIs) [60]. Aside from being present in an aggregated form in LBs and GCIs, duplications and triplications as well as point mutations in the *SNCA* gene encoding for αSYN have been associated with monogenetic familial forms of PD and DLB [12, 42, 65].

αSYN can adopt different conformations [33, 62], is enriched at the presynaptic terminals and thought to be involved in synaptic transmission and the recycling of synaptic vesicles [7, 55]. Under physiological conditions, αSYN is present as a soluble and natively unfolded monomer [57], probably in equilibrium with α-helical multimeric assemblies [6, 61]. During pathological conditions, αSYN monomers aggregate into high molecular weight β-sheet-rich oligomeric and fibrillar assemblies with each of these assemblies having unique functional and toxic properties [10, 13, 41, 53, 64].

The recently proposed prion-like behavior of αSYN implies that this amyloidogenic protein might act as a pathogenic factor spreading throughout the nervous system [23, 27]. Earlier indications promoting this idea originated from embryonic transplantation studies in PD patients [25, 27]. Along with these observations, direct cell-to-cell transmission of aggregated αSYN was confirmed in multiple experimental systems in cell culture and in vivo using fibrillar recombinant αSYN [15, 21, 23, 28]. The recent generation of pure fibrillar αSYN polymorphs with differences in structural and phenotypic traits has led to the hypothesis that different αSYN strains may be in part responsible for the heterogeneous nature of synucleinopathies [9]. In support of this hypothesis, we have previously shown that inoculation of two distinct recombinant αSYN assemblies, coined “Fibrils” and “Ribbons”, into rat substantia nigra (SN) resulted in remarkable differences in terms of behavior, neuronal integrity, αSYN pathology and spreading [36]. Subsequently, experiments using patient brain homogenates, fractionated or not, have provided more evidence that aggregated human αSYN can induce neurologic dysfunction when inoculated into the brain of model animals [31, 45, 63]. Indeed, αSYN present in GCI- and LB-enriched brain fractions was found to possess different biological features both in cells and in vivo*.* The distinct properties of aggregated αSYN were proposed to depend on both seed properties and the intracellular milieu [37]. The limitations of such studies lie within our inability to distinguish what aggregated αSYN per se and additional molecules, ranging from proteins to lipids, which either contaminate those αSYN-rich inclusions or are bound to pathogenic αSYN, trigger upon injection into the central nervous system (CNS) of animal models. To fill this gap and establish a structural-molecular basis for distinct synucleinopathies, we exploited the capacity of pathogenic αSYN aggregates present in the brain of patients suffering from PD, MSA and DLB to seed and template monomeric human αSYN in vitro via a protein misfolding cyclic amplification (PMCA) assay, resulting in pure αSYN fibrillar strains. Recent studies have also reported seeding and templating properties of pathogenic αSYN isolated from the brain or the cerebrospinal fluid of synucleinopathy patients using the same PMCA amplification method or alternatively real-time quaking-induced conversion (RT-QuIC) [11, 47, 52]. However, these studies were mainly focused on in vitro and cellular characterization. To date, a detailed in vivo comparison of pure αSYN assemblies derived from well-stratified patients with PD, MSA and DLB is still lacking.

Although protein aggregation appears to govern synucleinopathy progression, this does not entirely capture the complexity of the disease processes. The role of inflammatory processes within this intricate context, for example, has not been yet disentangled. Extracellular transmission of distinct αSYN strains could impact the immune tolerance and subsequent reaction, affecting neuronal integrity through the release of pro-inflammatory cytokines. As structurally distinct αSYN assemblies expose different polypeptide chains on their surfaces, they trigger helper and cytotoxic T cell responses in patients to different extents [22, 54], substantiating the genome-wide association study  (GWAS) link to specific major histocompatibility complex alleles [2].

Here, we compare side-by-side the properties of total human brain homogenates with in vitro amplified αSYN fibrillar assemblies. The capacity of these strains to aggregate in cell culture and to propagate and induce different pathologies and inflammatory responses in vivo upon inoculation in the rat substantia nigra pars compacta (SNpc) was investigated. We demonstrate that despite some heterogeneity between patients, both the homogenates and the PMCA-amplified strains reflect the characteristics of the different synucleinopathies with the MSA strains being the most aggressive and more similar to the PD-derived strains than to the DLB assemblies.

## Materials and methods

### Patient collection

Brain tissues from patients suffering from PD, MSA, and DLB as well as age-matched healthy controls were obtained at autopsy from four to six patients per condition through the UK Brain Bank (Imperial College London, UK). The clinicopathological description of the 19 patients is summarized in Table 1. After removal, the brains were hemisected using a mid-sagittal incision. One hemibrain was dissected fresh and the tissue blocks were frozen. The other hemibrain was formalin fixed for diagnostic neuropathological assessment. The extent of αSYN and tau pathology was assessed using Braak staging criteria and Thal amyloid phases were described [3, 4]. Brain regions enriched with αSYN aggregates were identified by histological examination, isolated and processed into brain homogenates. The cingulate cortex was isolated from the brains of the patients with PD and DLB. The cerebellum was obtained from the brains of the patients with MSA. Both regions were also extracted from the healthy control brains.

**Table 1:** Overview of the patients selected for the study

Case	N°	Sex	Age at onset	Duration	Genetics	Cause of death	Clinical diagnosis	Neuropathological diagnosis
				(years)				
PD 1	PD258	M	52	17	PARK2	Not reported	Tremulous parkinsonism with progression to PDD	LBD-Neocortical (αSYN, Braak VI); Tau Braak I, Thal phase 2
PD 2	PD341	M	54	14	/	Parkinson’s with dementia	Akinetic-rigid syndrome with progression to PDD, RBD	LBD-Neocortical (αSYN, Braak VI); Tau Braak II, Thal phase 2
PD 3	PD405	M	67	15	/	Not reported	Idiopathic Parkinson’s disease with dementia	LBD-Neocortical (αSYN, Braak VI); Tau Braak II, Thal phase ≥ 3
PD 4	PD523	F	68	18	/	Old age	Parkinson’s disease	LBD-Neocortical (αSYN, Braak VI); Tau Braak I, Thal phase ≥ 3
MSA1	PD043	M	78	8	/	Not reported	Atypical akinetic-rigid syndrome with prominent ataxia, PSP questioned	MSA; Tau Braak I, Thal phase 2
MSA2	PD080	M	65	6	PARK2	Parkinson’s disease	Akinetic-rigid syndrome with autonomic involvement and prominent ataxia. MSA suspected	MSA; Tau Braak I, Thal phase 0
MSA3	PD192	F	52	6	/	Bronchopneumonia	Akinetic-rigid syndrome with prominent bulbar/ autonomic involvement: MSA-P, RBD	MSA; Tau Braak I, Thal phase 0
MSA4	PD300	M	68	7	/	Pneumonia, MSA	Tremulous parkinsonism with prominent autonomic involvement: MSA/ Parkinson Plus syndrome suspected	MSA; Tau Braak I, Thal phase 0
MSA5	PD333	M	52	8	LRRK2	Respiratory failure, pneumonia	Parkinsonism, MSA questioned	MSA; Tau Braak I, Thal phase ≥ 3
MSA6	PD363	F	61	13	/	Aspiration pneumonia	MSA, bilateral pallidal DBS (with poor response)	MSA; Tau Braak I, Thal phase 1
DLB 1	PD163	M	70	6	/	Cerebrovascular disease, DLB	DLB, RBD	LBD-Limbic; Tau Braak I, Thal phase 2
DLB 2	PD330	F	71	5	/	DLB	DLB	LBD-Neocortical (αSYN, Braak VI); Tau Braak V, Thal phase ≥ 3
DLB 3	PD362	M	84	5	/	Not reported	Progressive cognitive decline (AD?) with abnormal gait	LBD-Neocortical (αSYN, Braak VI); Tau Braak VI, Thal phase ≥ 3
DLB 4	PD385	M	75	5	/	Not reported	DLB, RBD	LBD-Neocortical (αSYN, Braak VI); Tau Braak I, Thal phase ≥ 3
CTR 1	CO28	F	84	n.a	/	Pancreatic cancer	/	No αSYN; Tau Braak II, Thal phase 0
CTR 2	CO29	M	82	n.a	/	Metastatic liver and lung cancer	/	No αSYN; Tau Braak I, Thal phase 1
CTR 3	CO30	M	77	n.a	/	Cardiac failure/ prostate cancer	/	No αSYN; AGD, Thal phase 2
CTR 4	CO34	M	90	n.a	/	Respiratory failure/ bronchial cancer	/	No αSYN; Tau Braak I, Thal phase 0
CTR 5	CO53	F	89	n.a	/	Not reported	Hypertension, Type II diabetes, Hyperthyroidism, Previous CVA	No αSYN; Tau Braak II, Thal phase ≥ 3

The table summarizes the clinical history of the patients included in the study. Information about sex, age at onset, duration of the disease, genetics, cause of death, clinical and neuropathological diagnosis are described

*AD* Alzheimer’s disease, *AGD* argyrophilic grain disease, *αSYN* α-synuclein, *CVA* cerebrovascular accident, *DBS* deep brain stimulation, *DLB* dementia with Lewy bodies, *F* female, *LBD* Lewy body dementia, *LRRK2* leucine-rich repeat kinase 2, *M* male, *MSA* multiple system atrophy, *MSA-P* multiple system atrophy Parkinsonian subtype, *n.a.* not applicable, *PARK2* Parkinson’s disease associated gene 2, *PDD* Parkinson’s disease with dementia, *PSP* progressive supranuclear palsy, *RBD* rapid eye movement sleep behavior disorder

### Brain tissue homogenization

Frozen brain tissues were weighed in falcon tubes (15 or 50 ml depending on the total weight). The samples were diluted five times in PMCA buffer (150 mM KCl, 50 mM Tris–HCl pH 7.5) to obtain a homogenate at 20% (weight:volume). The homogenization was performed by sonication using the SFX 150 Cell Disruptor sonicator with a 3.17 mm microtip probe (Branson) for 1 min, with 10 s pulses followed by 10 s pauses in a biosafety level 3 environment (BSL-3). The homogenates were aliquoted and immediately frozen in liquid nitrogen before storage at − 80 °C. All contaminated surfaces were cleaned with SDS (1%) [16].

### Aggregated αSYN quantification in patients brain homogenates

Aggregated αSYN was quantified using a filter retardation assay and immunoblotting. To this aim, 0.5 mg of brain homogenates were immobilized on cellulose acetate membranes (0.2 μm pore size, Millipore Corp., Bedford, MA) by filtration using a 48-slot slot-blot filtration apparatus (GE Healthcare). The membranes were blocked in 5% dried skimmed milk and probed with either the 4B12 (Biolegend, cat # 807801) and P-S129 αSYN antibody (mouse 11A5, provided by Elan Pharmaceuticals, Inc., Dublin, Ireland). Following wash with tris buffered saline with triton (TBST), the membranes were incubated with horseradish peroxidase (HRP) conjugated goat-anti-mouse IgG3 secondary antibody (Thermo, cat #M32707) for 1 h at room temperature. Proteins were visualized using ECL reagents (Pierce, USA).

The Cisbio fluorescence resonance energy transfer (FRET) assay (Cisbio, France, cat # 6FASYPEG) was also used to quantify aggregated αSYN in patients’ brain homogenates, following the manufacturer’s recommendations. Briefly, patient brain homogenates were diluted to 2.5% (W:V) in lysis buffer provided in the HTRF kit. 10 µl of each diluted brain homogenates were loaded into a 96 well plate and mixed with 10 µl of the FRET donor and acceptor antibodies in the kit. The plate was sealed with a film (CmlAB, Danemark, cat#13076-9P-500) and incubated for 20 h at 20 °C without shaking in a Thermomixer comfort (Eppendorf, Montesson, France). After incubation, time-resolved FRET was measured upon excitation at 337 nm using a plate reader (CLARIOstar, BMG Labtech, Germany) [14]. The HTRF signal was recorded at two different wavelengths (665 nm and 620 nm). The amount of aggregated αSYN was derived from the 665/620 nm fluorescence ratio and multiplied by 10.000.

### Protein misfolding cyclic amplification assay

All operations were performed in BSL-3. Brain homogenates were diluted in PMCA buffer (150 mM KCl, 50 mM Tris–HCl, pH 7.5) containing monomeric αSYN (100 µM) to a final concentration of 2% (W:V), equivalent to 6 mg of brain tissue, as described previously for other tissues [17]. The sample was split in two tubes of PCR strips (BIOplastics, Landgraaf, The Netherlands). PMCA amplification was performed in quadruplicates for each patient using the Q700 generator and a 431MPX horn (Qsonica, Fisher scientific, Illkirch, France). The power of the horn was set to 30% of maximal amplitude. The program of amplification consisted in 15 s of sonication and 5 min pause at 31 °C. Every hour, 5 µl were withdrawn from each tube and diluted in 300 µl of 10 µM of Thioflavin T. The amplification was monitored by measuring Thioflavin T fluorescence using a Cary Eclipse Fluorescence Spectrophotometer (Agilent, Les Ulis, France) with fixed excitation and emission wavelength at 440 nm and 480 nm respectively. Cycle 2, 3 and 4 were performed following the same protocol using 1% of the preceding cycle reaction as seeds for PD and DLB cases, 5% for MSA cases. The amounts of brain homogenates and PMCA amplified assemblies used in each amplification reaction were defined through an optimization study aimed at maintaining high stringency by minimizing the de novo aggregation of αSYN under our experimental conditions. The time at which an aliquot from one amplification reaction was withdrawn for a subsequent amplification reaction was also defined through an optimization study aimed at avoiding de novo αSYN fibrillar assemblies formation.

At cycle 4, PMCA reactions products were spun for 30 min at 50,000*g*, the amount of monomeric αSYN in the supernatant was assessed spectrophotometrically and the pelleted assemblies were resuspended in phosphate-buffered saline (PBS) buffer. All resuspended assemblies, at a final concentration of 100 µM αSYN, were fragmented prior to in vivo use by sonication for 20 min in 2-ml Eppendorf tubes in a Vial Tweeter powered by an ultrasonic processor UIS250v (250 W, 2.4 kHz; Hielscher Ultrasonic, Teltow, Germany), aliquoted, flash frozen in liquid nitrogen and stored until use at − 80 °C.

### Proteolytic digestion

De novo assembled αSYN fibrils and ribbons as well as PD, MSA and DLB patients PMCA-amplified αSYN assemblies (1.4 mg/ml) in 150 mM KCl, 50 mM Tris–HCl, pH 7.5, 1 mM EGTA were treated at 37 °C by Proteinase K (3.8 µg/ml) (Roche). Aliquots were removed at different time intervals following addition of the protease and transferred into Eppendorf tubes maintained at 90 °C containing sample buffer (50 mM Tris–HCl, pH 6.8, 4% SDS, 2% beta-mercaptoethanol, 12% glycerol and 0.01% bromophenol blue) to arrest immediately the cleavage reaction. After incubation of each tube for 5 min at 90 °C, the samples were processed to monitor the time course of αSYN cleavage by polyacrylamide gel electrophoresis (PAGE) (15%) after staining with Coomassie blue.

### Transmission electron microscopy

The morphology of the de novo assembled and PMCA-amplified αSYN assemblies was assessed by transmission electron microscopy (TEM) in a Jeol 1400 transmission electron microscope following adsorption onto carbon-coated 200 mesh grids and negative staining with 1% uranyl acetate. The images were recorded using a Gatan Orius CCD camera (Gatan).

### Conformational characterization using FILA4 antibody

αSYN fibrils, ribbons assembled de novo and PMCA-amplified αSYN assemblies (0.2 µg) in 150 mM KCl, 50 mM Tris–HCl, pH 7.5, were spotted onto single nitrocellulose membranes. The membranes were blocked in 5% dried skimmed milk and probed with the aggregated αSYN specific antibody FILA4 at a concentration of 0.28 µg/ml [35]. Following wash with TBST, the membranes were incubated with HRP-conjugated swine-anti-rabbit secondary antibody (DAKO, Copenhagen, Denmark) for 1 h at room temperature. Proteins were visualized using ECL reagents (Pierce, USA). ECL signal was detected using a ChemiDocTM MP (BioRad) and the protocol “Chemi”. Acquisition was performed using the protocol ‘Signal Acquisition Mode” from 1 to 60 s of exposure. Images were processed and quantified using Image Lab. The FILA4 signal intensity for each spot was integrated using “Lanes & Bands” function.

### αSYN seeding assay

Human H4 neuroglioma cells stably expressing αSYN-YFP fusion protein were seeded at 6000 cells per well in a 384-well plate. Cells were incubated with patient-derived homogenates or PMCA-amplified αSYN assemblies (175 nM). Recombinant αSYN fibrils (175 nM) were used as a positive control. The particle concentration of patient-derived or de novo assembled fibrillar αSYN was obtained by dividing αSYN monomeric concentration by the average number of molecules (as measured by analytical ultracentrifugation (AUC) and TEM). The average number of molecules measured for all amplified αSYN fibrils was 8300 ± 300. The average length of all amplified fibrils was 41 ± 2 nm. Cells were fixed in 10% paraformaldehyde (PFA) 24 h after incubation with the different brain homogenates or PMCA-amplified αSYN assemblies and were conserved in PBS with DAPI (4′,6-diamidino-2-fenylindool, 1/5000) until analysis. The number of αSYN aggregates and PαSYN positive cells was quantified based on YFP or PαSYN fluorescence respectively and the number of cells was determined with DAPI staining using the Operetta High Content Imaging System (PerkinElmer) and Harmony 4.9 software. The width to length ratio and the roundness was determined using the same software. The detailed photographs were taken by a Zeiss LSM 880 confocal microscope.

### Primary cortical neuron cultures

Primary cortical neurons were prepared from the cortex of E16 FVB/N mouse embryos. Meninges were removed, cortices were dissected, and subsequently dissociated according to the protocol. Neurons were plated in a 24-well plate with coverslips at a density of 200,000 cells per well. Cells were treated with PMCA-amplified αSYN assemblies (175 nM) in Neurobasal medium supplemented with 2 mM L-glutamine and 2% B27 at DIV 1. Cells were fixed in 10% PFA at DIV 7 for immunocytochemical analysis.

### Recombinant AAV production and purification

Vector production and purification was performed as previously described [39]. The plasmids include the constructs for the AAV7 serotype, the AAV transfer plasmid encoding the human mutant A53T αSYN under the control of the ubiquitous CMVie enhanced synapsin1 promoter and the pAdvDeltaF6 adenoviral helper plasmid. Real-time PCR analysis was used for genomic copy determination.

### Stereotactic injections

All animal experiments were carried out in accordance with the European Communities Council Directive of November 24, 1986 (86/609/EEC) and approved by the Bioethical Committee of the KU Leuven (ECD project P067-2013 and P085-2014, Belgium). Eight-week-old female Sprague Dawley rats (Janvier, France) weighing about 200 to 250 g were housed under a normal 12-h light and/or dark cycle with free access to pelleted food and tap water. All surgical procedures were performed using aseptic techniques. Rats were anaesthetized with ketamine (60 mg/kg, intraperitoneal (i.p.), Ketalar, Pfizer, Belgium) and medetomidine (0.4 mg/kg, i.p., Dormitor, Pfizer, Belgium). Following anesthesia, the rodents were placed in a stereotactic head frame (Stoelting, IL, USA). Injections were performed with a 30-gauge needle (VWR International, Haasrode, Belgium) and a 10-µl Hamilton syringe (Hamilton, Bonaduz, GR, Switzerland). Animals were stereotactically injected into the SN with 2.5 µl of total brain homogenate (20% W:V) or PMCA-product (100 µM) supplemented with 0.5 µl of A53T αSYN recombinant adeno-associated viral vector 2/7 (rAAV2/7 vector) (5.4E + 11 GC/ml vector dose) or PBS. rAAV control animals were injected with 3 µl of diluted A53T αSYN rAAV2/7 vector in the SN (equal vector dose). Stereotactic coordinates used for the SN were anteroposterior, − 5.3; lateral, − 2.0; and dorsoventral, − 7.2 calculated from the dura using bregma as reference.

To determine the overall amount of aggregated αSYN using the HTRF Cisbio assay in the brains of animals after inoculation with the different PMCA-amplified αSYN assemblies, 5 µl of PMCA-product (100 µM) supplemented with 0.5 µl of A53T αSYN rAAV2/7 vector (5.4E + 11 GC/ml vector dose) were injected over three injection sites in the rat striatum (STR). Stereotactic coordinates used for the STR were anteroposterior, 0.0; lateral, − 2.0; and dorsoventral, − 5.5/− 4.5/− 4.0 calculated from the dura using bregma as reference. The injection rate was 0.25 µl/min and the needle was left in place for an additional five minutes before being retracted.

### Cylinder test

The cylinder test was used to quantify forelimb use. Contacts made by each forepaw with the wall of a 20-cm-wide clear glass cylinder were scored from the videotapes by an observer blinded to the animal’s identity. A total of 25 contacts were recorded for each animal. The number of impaired forelimb contacts was expressed as a percentage of total forelimb contacts. Non-Lesioned control rats should score around 50% in this test.

### Immunohistochemical stainings

Rats were sacrificed with an overdose of sodium pentobarbital (200 mg/kg, i.p., Dolethal, Vetoquinol, Belgium) followed by intracardial perfusion with 4% PFA in PBS. After post-fixation overnight in 4% PFA, 50 µm-thick coronal brain sections were made with a vibrating microtome (HM 650 V, Microm, Germany). IHC was performed on free-floating sections using antibodies against tyrosine hydroxylase (TH, rabbit polyclonal, Ab152, 1:5000, Merck Millipore, Massachusetts, US), phosphorylated αSYN (P-S129 αSYN, mouse 11A5, 1:5000, provided by Elan Pharmaceuticals, Inc., Dublin, Ireland and rabbit, Ab51253, 1:5000, Abcam, Cambridge, UK), Iba1 (goat polyclonal, Ab107159, 1:1000, Abcam), MHC Class II (mouse, MCA46G, 1:250, Serotec), CD4 (mouse, MCA55GA, 1:500, Serotec) and CD8 (mouse, MCA48GA, 1:500, Serotec). Sections were pretreated with 3% hydrogen peroxide for 10 min and incubated overnight with primary antibody in 10% swine, normal goat or rabbit serum (DakoCytomation, Belgium). As secondary antibody (DakoCytomation, Belgium) we used biotinylated anti-rabbit IgG (TH, 1:600), anti-mouse IgG (P-S129 αSYN, 1:600; MHC Class II, CD4 and CD8, 1:300) and anti-goat IgG (Iba1, 1:300), followed by incubation with streptavidin-horseradish peroxidase complex (1:1000, DakoCytomation, Belgium). TH immunoreactivity was visualized using Vector SG (SK-4700, Vector Laboratories, CA) and P-S129 αSYN, Iba1, MHC Class II, CD4 and CD8 immunoreactivity were visualized using 3,3-diaminobenzidine (0.4 mg/ml, Sigma-Aldrich) as a chromogen.

### Immunofluorescent stainings

Sections were rinsed three times in PBS and then incubated overnight in PBS-0.1% triton X-100 with 10% donkey serum (Jackson ImmunoResearch Laboratories, Inc., UK) and the following antibodies: mouse anti-P-S129 αSYN (11A5, 1:5000, Elan Pharmaceuticals), chicken anti-TH (THY, 1:1000, Aves) and rabbit anti-GST-π (312, 1:1000, MBL Life Science), mouse anti-P-S129 αSYN (11A5, 1:5000, Elan Pharmaceuticals), rabbit anti-Olig2 (Ab9610, 1:1000, Merck Millipore) or mouse anti-DARPP-32 (H3, Santa Cruz Biotechnology 1:500). After three rinses in PBS-0.1% triton X-100 the sections were incubated in the dark for 2 h in fluorochrome-conjugated secondary antibodies: donkey anti-mouse Alexa 488 (1:1000, Molecular Probes™, Invitrogen, Belgium), donkey anti-chicken Cy3 (1:500, Jackson ImmunoResearch) and donkey anti-rabbit Alexa 647 (1:1000, Molecular Probes™, Invitrogen) or donkey anti-mouse Alexa 488 (1:1000, Molecular Probes™, Invitrogen, Belgium) and donkey anti-rabbit Alexa 647 (1:1000, Molecular Probes™, Invitrogen). After being rinsed in PBS and mounted, the sections were coverslipped with Mowiol 4–88 (Calbiochem®, California, US) and DAPI (1:1000).

For immunocytochemical analysis, fixed coverslips were incubated overnight in PBS-0.1% triton X-100 with 10% donkey serum (Jackson ImmunoResearch Laboratories, Inc., UK) and primary antibodies against mouse P-S129 αSYN (11A5, 1:5000, Elan Pharmaceuticals) and rabbit β3-tubulin (Ab52901, 1:1000, Abcam). The secondary antibodies used were donkey anti-mouse Alexa 488 (1:1000, Molecular Probes™, Invitrogen, Belgium) and donkey anti-rabbit Alexa 647 (1:1000, Molecular Probes™, Invitrogen, Belgium). Fluorescent stainings were visualized by confocal microscopy with an LSM 880 unit (Zeiss, Belgium).

### Stereological quantification

The number of TH- and P-S129 αSYN  positive cells in the SN was determined by stereological measurements using the Optical fractionator method in a computerized system as described before [5] (StereoInvestigator; MicroBright-Field, Magdeburg, Germany). Every fifth section throughout the entire SN was analyzed, with a total of seven sections for each animal. The coefficients of error, calculated according to the procedure of Schmitz and Hof as estimates of precision [46], varied between 0.05 and 0.10. All the analyses were performed by an investigator blind to different groups.

### Striatal immunoreactivity quantification

Seven sections covering the whole STR were stained using an antibody against TH as previously described. Images were acquired using the LEICA DM6B optical microscope (Leica, Wetzlar, Germany) with a Leica DFC 7000 T digital camera (Leica) and the Leica Application Suite software (Leica). Intensity measurement was performed using the software ImageJ.

### Evaluation of the spreading of αSYN pathology

Spreading of the αSYN pathology was analyzed and scored by immunohistochemical analysis using an antibody against P-S129 αSYN as previously described. Data are shown as heat maps to illustrate the distribution of the αSYN pathology throughout seven different brain regions in a semi-quantitative manner. The selected brain regions are the following: (1) and (2) adjacent sections of caudate putamen (CPu) (respectively bregma 2.16; bregma 1.20); (3) lateral caudate putamen (CPu) and internal capsule (ic) (bregma − 1.80); (4) SNpc and cerebral peduncle (cp) (bregma − 4.36); (5) dorsal tier of substantia nigra pars compacta (SNpc) and pars reticulata (SNpr) (bregma − 5.28, injection site); (6) medial and ventral tier of SNpc (bregma − 5.76) and (7) lateral SNpc and SNpr (bregma − 6.24).

## Results

### In vitro characterization of patient-derived αSYN strains

**Fig. 1:**
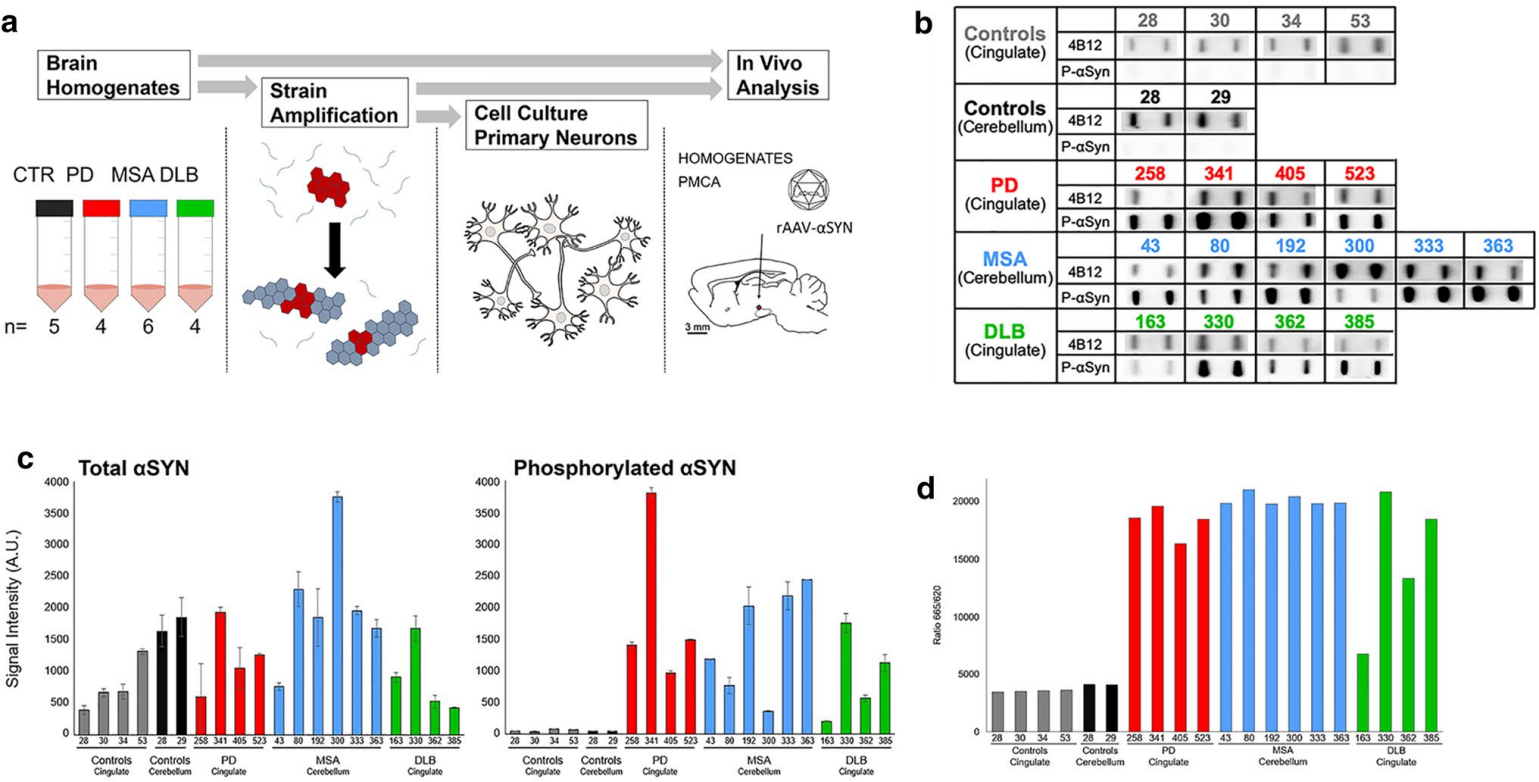
Quantification of aggregated αSYN in patients' brains. **a** Schematic representation of the experimental setup. Brain tissues from patients suffering from PD, MSA and DLB as well as age-matched healthy controls (*n* = 4–6) were obtained at autopsy and processed into total brain homogenates. The pathogenic αSYN aggregates present in the patient’s brain were utilized to seed and template monomeric human αSYN in vitro via a PMCA assay. Total brain homogenates and in vitro amplified αSYN fibrillar assemblies were carefully compared upon administration to cell culture and upon inoculation in the rat brain. **b** The amount of pathogenic, aggregated αSYN in the different brain homogenates was determined using a filter retardation assay. 50 µl of brain homogenates (1% in PBS, W:V), equivalent to 0.5 mg total proteins, were filtered on cellulose acetate membranes and probed with 4B12 (total αSYN) or anti-phosphorylated αSYN (PαSYN). **c** Quantification of total αSYN and PαSYN in the different brain homogenates presented in panel b. **d** The amount of pathogenic, aggregated αSYN in the different brain homogenates (2.5% in PBS, weight:volume) was quantified using the Cisbio FRET assay as described in the “Materials and methods” section

To investigate the presence of distinct human αSYN strains in vivo we analyzed brain specimens from four to six deceased patients carrying the clinical and neuropathological diagnosis of PD, DLB or MSA, as well as five age-matched control patients (CTR). The clinicopathological description of the 19 patients is summarized in Table 1. LB-rich regions were selected, e.g. the cingulate cortex for PD and DLB and the cerebellum for MSA patients, which were subsequently processed into crude homogenates (Fig. 1a). The amount of aggregated αSYN and αSYN phosphorylated at Ser129 (PαSYN) was assessed by filter trapping (Fig. 1b). The amount of αSYN and PαSYN trapped on the cellulose acetate membranes varied significantly amongst PD, MSA or DLB patients (Fig. 1b, c), but was significantly higher in PD, DLB or MSA than in control patient brain homogenates. Very low levels, if any, of aggregated PαSYN was detected in control patients (Fig. 1b, c). The amount of aggregated αSYN was also quantified using the HTRF Cisbio assay [14]. Similar to what was observed using the filter trap assay, the amounts of aggregated αSYN in PD, DLB or MSA patient brain homogenates were significantly higher than those in control patients (Fig. 1d).

**Fig. 2:**
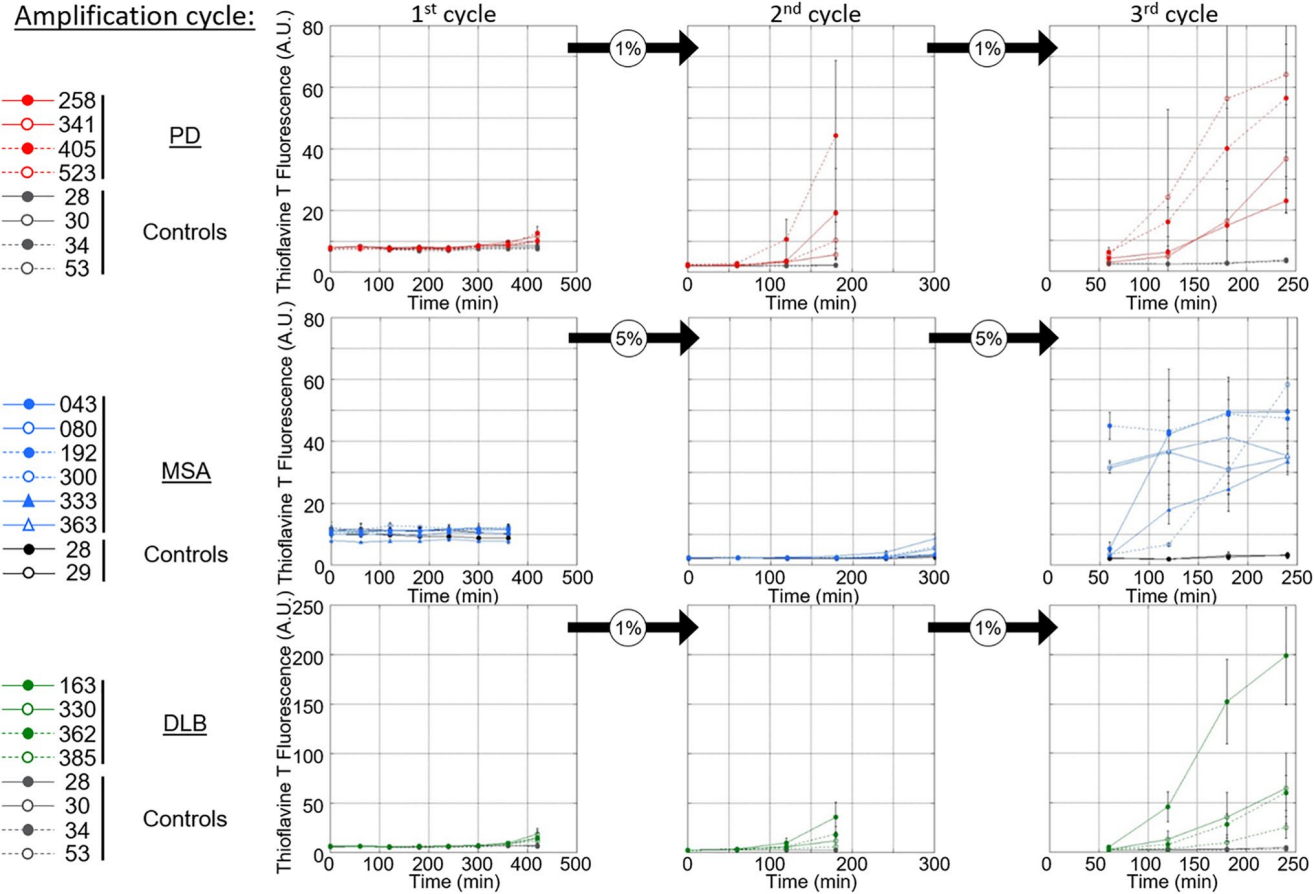
Amplification of pathogenic αSYN from PD, MSA and DLB patients by PMCA. PMCA was performed on human brain homogenates (2% (W:V) for the 1st cycle, the indicated amounts (V:V) for the next cycles, in PMCA buffer containing monomeric αSYN, 100 µM) from PD (top, in red), MSA (middle, in blue), DLB (bottom, in green) and the corresponding control (in black or gray) patients. The amounts of brain homogenates and PMCA- amplified assemblies used in each amplification reaction were defined through an optimization study aimed at maintaining high stringency by minimizing the de novo aggregation of αSYN under our experimental conditions. The time at which an aliquot from one amplification reaction was withdrawn for a subsequent amplification reaction was also defined through an optimization study aimed at avoiding the formation of de novo of αSYN fibrillar assemblies. The curves represent an average of four replicates ± SD

**Fig. 3:**
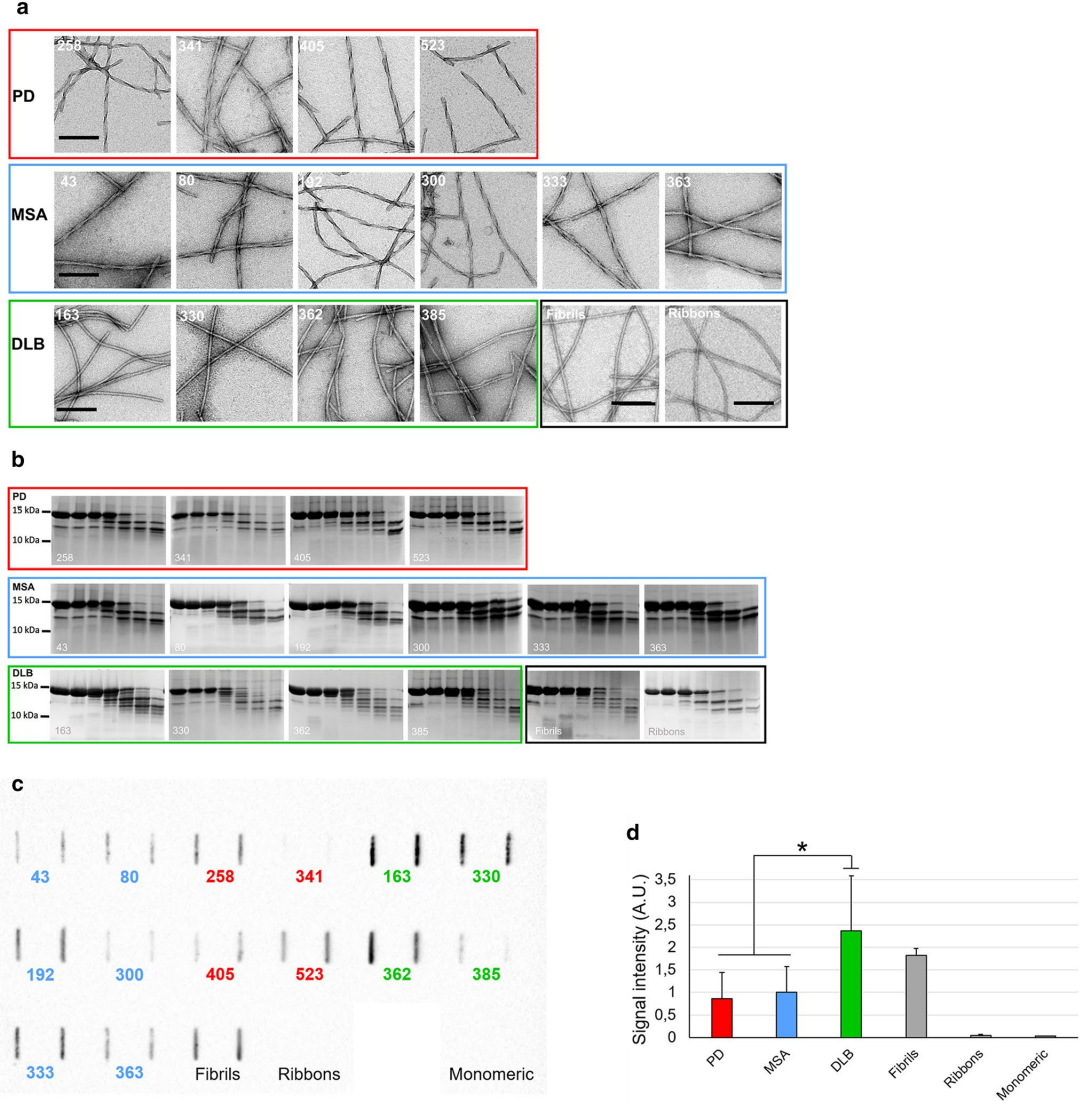
In vitro characterization of patient-derived αSYN assemblies. **a** Electron micrographs of αSYN assemblies obtained after the 3rd cycle of amplification by PMCA from PD, MSA and DLB patients and from de novo generated αSYN Fibrils and Ribbons. Scale bar = 200 nm. **b** Limited proteolytic patterns of αSYN assemblies obtained after the 3rd cycle of amplification by PMCA from PD, MSA and DLB patients and from de novo generated αSYN Fibrils and Ribbons. Monomeric αSYN concentration is 100 µM. Proteinase K concentration is 3.8 µg/ml. Samples were withdrawn from the reaction before PK addition (lane most to the left), immediately after PK addition (second lane from left) and at time 1, 5, 15, 30 and 60 min from left to right in all panels. PAGE analysis was performed as described in the “Materials and methods” section and the gels were stained with Coomassie blue. The molecular weight markers are shown on the left. **c** The conformational FILA4 antibody distinguishes equal amounts (0.2 µg) of PMCA-amplified αSYN strains spotted on a single nitrocellulose membrane. **d** Quantification of FILA4 signal for PD, MSA, DLB, and reference samples (Fibrils, Ribbons, and monomeric αSYN). Data represent mean ± SE (*p < 0.05 for one-way ANOVA, *n* = 4–6 PD- or MSA-amplified strains versus DLB-amplified strain)

We next used a PMCA assay we implemented to template monomeric αSYN assemblies into pathogenic aggregates that reproduce the structural characteristics of aggregated αSYN in patient brains [17]. Amplification was performed in an iterative manner to ensure that monomeric αSYN does not assemble into fibrils de novo under our experimental conditions. The seeded assembly of monomeric αSYN (100 µM) in the presence of diseased brain homogenates (2% in assembly buffer) was monitored using Thioflavin T binding (Fig. 2, left panels). After 6 h, the aggregation solution was diluted (20-fold for MSA patients, 100-fold for PD and DLB patients) in assembly buffer containing 100 µM monomeric αSYN under which the aggregation reaction was further monitored (Fig. 2, central panels). A third round of templating was initiated by diluting the fibrillar assemblies generated (after 5 h for MSA patients, and 3 h for PD and DLB patients) in assembly buffer containing monomeric αSYN (Fig. 2, right panels). Control brain homogenates did not exhibit any seeding potential under the stringent experimental conditions (Fig. 2). The αSYN fibrillar assemblies were imaged by TEM and their limited proteolytic patterns upon exposure to Proteinase K were analyzed by SDS-PAGE. TEM analysis revealed disease-specific differences in the shape of the fibrils. PD and MSA patient-derived αSYN fibrils obtained by PMCA exhibited a relatively flat and twisted appearance, significantly resembling the fibrillar polymorph “Ribbons” (Fig. 3a). Those derived from DLB patients were cylindrical, exhibiting no twists and indistinguishable from the fibrillar polymorph “Fibrils” (Fig. 3a). Furthermore, PMCA-derived PD and MSA αSYN fibrils exhibited similar limited proteinase K degradation patterns that differed from those derived from DLB patients (Fig. 3b). Limited proteolysis patterns of PD and MSA patient-derived αSYN fibrils resembled that of the fibrillar polymorph “Ribbons” while the proteolysis pattern of DLB patient-derived αSYN fibrils was similar to the fibrillar polymorph “Fibrils” (Fig. 3b). Last, we further characterized the PMCA-amplified αSYN assemblies using the conformational antibody FILA4. All PMCA-amplified patient-derived αSYN fibrils were recognized by the aggregated αSYN conformational antibody FILA4 to a different extent (Fig. 3c). In line with our results generated by TEM and limited Proteinase K degradation, PMCA-derived PD and MSA αSYN fibrils were conformationally distinguishable from patient-derived DLB fibrils as significantly lower FILA4 signal was detected in those samples (Fig. 3d). We conclude from these observations that fibrils derived from patients developing one given synucleinopathy have comparable fingerprints and morphology. We further conclude that PD and MSA patient-derived fibrils have a similar shape and limited proteolysis pattern, distinct from that of DLB patient-derived fibrils.

### PD, MSA and DLB patient-derived fibrillar αSYN assemblies induce the aggregation of endogenous αSYN in cell culture

**Fig. 4:**
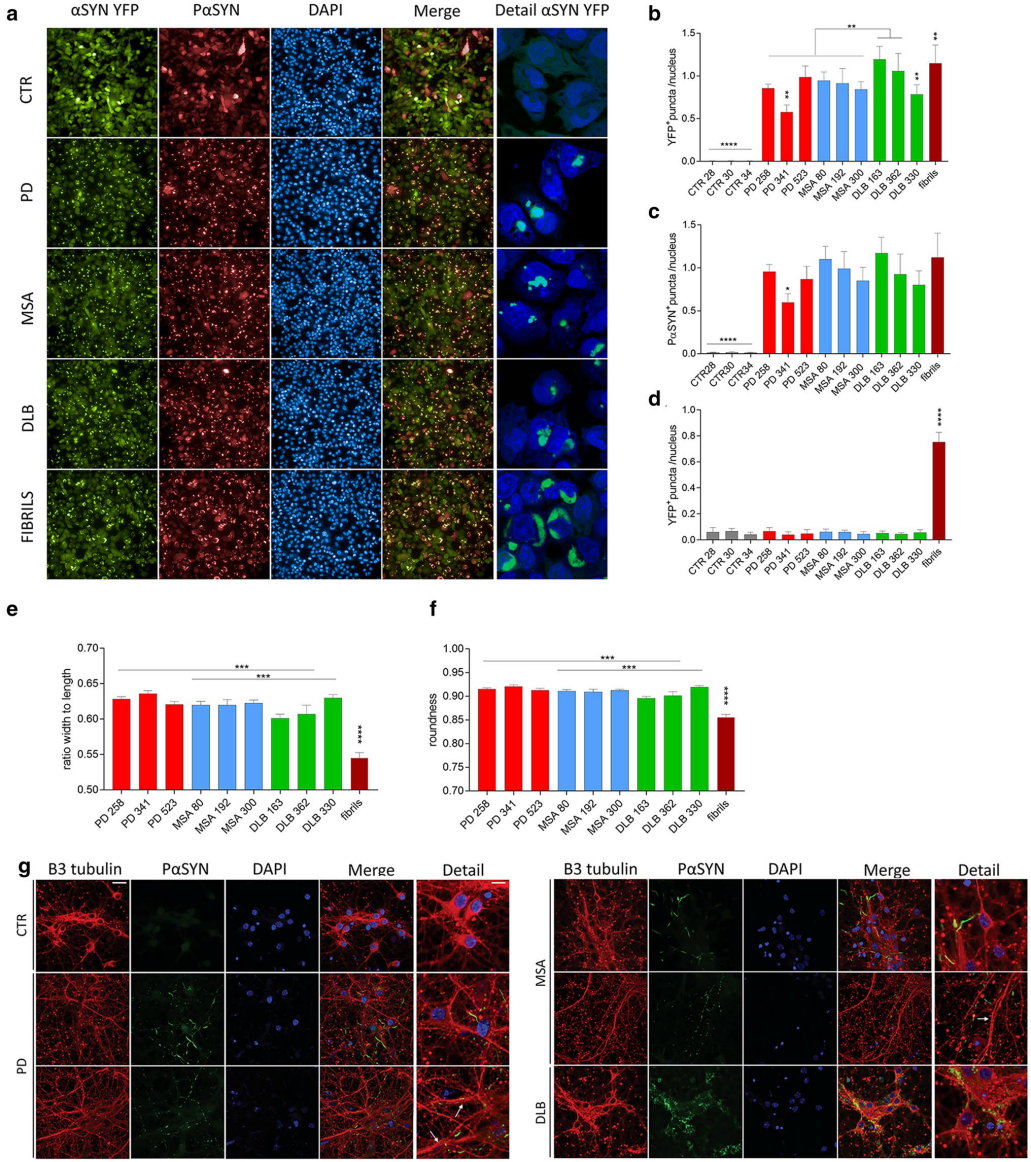
Characterization of patient-derived αSYN strains in cellular models. **a** Representative photomicrographs of the human neuroglioma (H4) cells stably expressing αSYN-YFP upon administration of the different PMCA-amplified αSYN strains (175 nM; green) or recombinant αSYN fibrils (175 nM; green) stained for PαSYN (red). DAPI was used to visualize the nuclei (blue) (left panel). Detailed photomicrographs of the YFP^+^ puncta present in the H4 cells upon administration of the different PMCA-amplified αSYN strains (right panel). **b** The number of YFP^+^ puncta per nucleus in H4 cells (presented in panel a) was quantified 24 h after incubation with the different PMCA-amplified αSYN strains. **c** The number of PαSYN^+^ puncta per nucleus in H4 cells (presented in panel a) was quantified 24 h after incubation with the different PMCA-amplified αSYN strains. **d** The number of YFP^+^ puncta per nucleus in H4 cells was quantified 24 h after incubation with total brain homogenates. **e** The width to length ratio in H4 cells was measured 24 h after incubation with the different PMCA-amplified αSYN strains. **f** The roundness in H4 cells was measured 24 h after incubation with the different PMCA-amplified αSYN strains. Results shown as mean ± SEM (**p < 0.01, ***p < 0.001, ****p < 0.0001 for one-way ANOVA with Tukey’s post-hoc analysis, *n* = 3 patients per condition). **g** Representative photomicrographs of the primary cortical neurons after incubation with the different PMCA-amplified αSYN strains for 7 days stained for the neuronal marker β3-tubulin (red) and for PαSYN (green). DAPI was used to visualize the nuclei (blue). This experiment was repeated three times. Scale bar represents 10 μm

We next assessed the seeding capacity of the distinct PMCA-amplified αSYN fibrillar strains in a human H4 neuroglioma cell line expressing αSYN-YFP. Patient-derived or de novo assembled fibrillar αSYN were fragmented to generate fibrillar particles with an average size of 42–52 nm that are suitable for endocytosis, as described in the “Materials and methods” section. There was no difference in the average size of aggregates for the different diseases. The particle concentration of patient-derived or de novo assembled fibrillar αSYN was assessed by AUC and was quantitatively analyzed by TEM as previously described [41, 48, 49]. Recombinant αSYN fibrils were used as a positive control. Exposure of H4 cells to all αSYN strains for 24 h led to αSYN aggregation as quantified by the number of YFP^+^ puncta per H4 cell nucleus (Fig. 4a, b). No such aggregation was observed upon exposure of H4 cells to CTR samples (Fig. 4a, b) or brain homogenates (Fig. 4d). Aggregated αSYN-YFP co-localized to a large extent with PαSYN aggregates (Fig. 4c). Recombinant fibrils induced the highest number of YFP^+^ puncta and gave rise to large crescent-shaped YFP^+^ PαSYN^+^ aggregates in H4 cells (Fig. 4a, b). When the patients were grouped per disease, the DLB group showed most inclusions, followed by the MSA patients and then the PD group (Fig. 4b). Analysis per patient revealed that the number of YFP^+^ puncta was significantly higher in αSYN strains derived from two DLB patients (DLB163 and DLB 362) compared to all the other experimental conditions (PD, MSA and DLB 330). Human H4 cells incubated with αSYN strains from PD 341 exhibited less YFP^+^ aggregates in contrast to the two other PD patients (PD 258 and 523). Next, we assessed the morphology of the aggregates. H4 cells incubated with recombinant αSYN fibrils presented inclusions with a significantly reduced width to length ratio and roundness compared to the patient-derived material. In addition, the inclusions from the DLB group displayed a slightly reduced width to length ratio and roundness when compared to the PD and MSA conditions (Fig. 4e, f). Taken together, these data demonstrate that the distinct PMCA-amplified αSYN strains seed monomeric αSYN aggregation in cell culture towards a different extent. Exposure of primary cortical neurons for 7 days to the different PMCA-amplified αSYN fibrillar strains, but not control samples, led to the formation of phosphorylated αSYN deposits (Fig. 4g). Interestingly, we observed a distinct phosphorylation pattern for the different PMCA-amplified αSYN fibrillar strains. Neurons exposed to MSA and PD patient-derived αSYN strains presented two morphologically distinct types of PαSYN-positive neurites, namely thicker linear neurites (top panel) and neurites containing a dotted phosphorylation pattern (lower panel). Those incubated with αSYN strains derived from DLB patients, however, displayed diffuse PαSYN deposits within the soma, surrounding the nucleus. In addition, we also observed differences in β3 tubulin staining among the experimental conditions. Neurons treated with PD and MSA αSYN strains presented larger dystrophic neurites compared to the CTR or DLB αSYN strains, indicative of ongoing neurodegeneration (Fig. 4g detail). These findings further suggest that distinct PMCA-amplified αSYN strains seed the aggregation of endogenous αSYN to different extents and induce different phenotypic changes.

### Intracerebral inoculation of PD, MSA and DLB patient-derived αSYN strains trigger changes in motor behavior

**Fig. 5:**
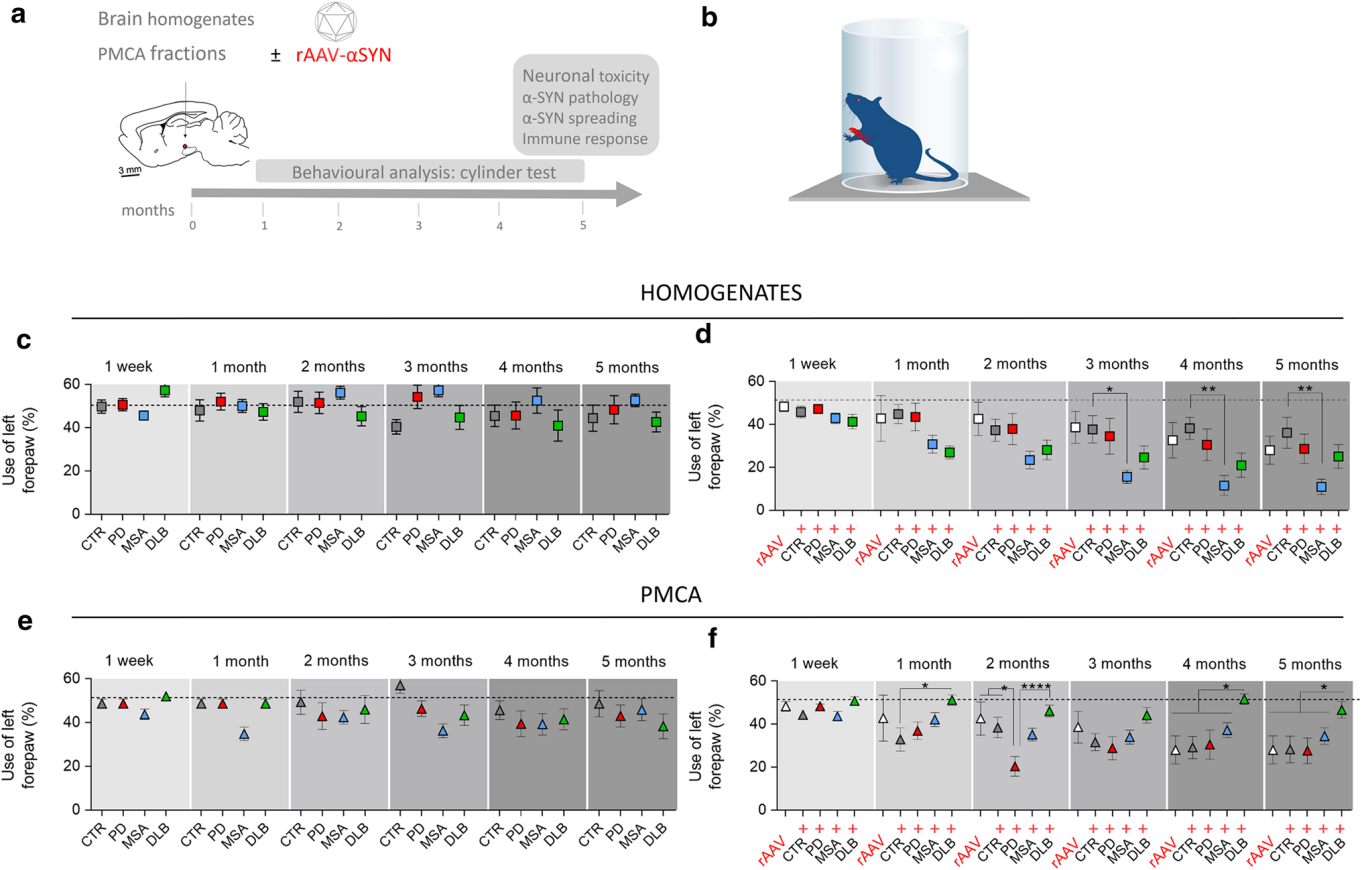
Assessment of motor deficits after intracerebral inoculation of the different patient-derived homogenates and PMCA-amplified αSYN strains. **a** Schematic representation of the experimental set-up. Rats were inoculated with the different patient-derived brain homogenates or PMCA-amplified αSYN strains in absence or presence of human αSYN expression. Animals were subjected to the cylinder test on a monthly basis. The in vivo behavior of the distinct human αSYN strains was characterized by assessing neuronal toxicity, αSYN pathology, spreading of αSYN pathology and inflammatory response at the final time point of 150 days. **b** Schematic drawing illustrating the set-up of the cylinder test to assess motor behavior. **c**, **d** Alterations in motor behavior after inoculation of PD, MSA, DLB patient-derived homogenates and age-matched controls in absence (**c**) or presence of rAAV2/7-mediated overexpression of αSYN (rAAV) (**d**) were assessed using the cylinder test. **e**, **f** Alterations in motor behavior after inoculation of PD, MSA or DLB-derived PMCA-amplified αSYN assemblies in absence (**e**) or presence of rAAV-αSYN overexpression (**f**) were assessed using the cylinder test. Results shown as mean ± SEM (*p < 0.05, **p < 0.01, ****p < 0.0001 for two-way ANOVA with Tukey’s post-hoc analysis, *n* = 4–6 patients per group and *n* = 3 per patient). A total of 12–16 animals per group was included in this study

To determine whether the distinct characteristics of αSYN strains reflect in vivo, we inoculated brain homogenates from PD, MSA and DLB patients in parallel with pure PMCA-amplified αSYN assemblies derived from those patients into the rat SN in absence or presence of a rAAV2/7 vector encoding human αSYN (Fig. 5a). A total of 12–16 animals per group was included in this study, representing 4–6 patients per condition and 3 animals per patient. We have previously demonstrated that rAAV-mediated expression of αSYN in the rodent brain induces time-dependent neuropathological changes reminiscent of Lewy pathology [40]. In addition, a more robust model was generated by the combination of rAAV-αSYN injection into the brain with recombinant protein, resulting in enhanced neurodegeneration and motor behavior, as well as more pronounced Lewy body-like inclusions and protein spreading [36]. To monitor behavioral performance, we subjected the animals to the cylinder test on a monthly basis for five months (Fig. 5b). Neither the brain homogenates nor the PMCA-amplified assemblies induced significant motor deficits in naive animals within the experimental time frame (Fig. 5c, e). When we combined patient-derived material with rAAV-mediated αSYN expression, clear motor deficits developed progressively over time from 3 months post injection and onwards. The MSA patient brain homogenates induced the most prominent motor deficits compared to the CTR patients (MSA, 11 ± 4% remaining forepaw use versus CTR, 36 ± 7%, Fig. 5d). Animals injected with rAAV-αSYN alone or together with PMCA-amplified αSYN assemblies derived from CTR, PD or MSA patients presented similar motor deficits (Fig. 5f). Surprisingly the animals injected with PMCA-amplified αSYN assemblies derived from DLB patients did not display detectable motor deficits. To rule out any technical issues we inoculated an additional group of animals with PMCA-amplified αSYN assemblies derived from DLB and MSA patients (*n* = 8–12 animals per condition) and similar results were obtained (data not shown). We conclude from these findings that rats robustly develop motor deficits only when brain homogenates from PD, MSA and DLB patients, and to a much lesser extent PMCA-amplified αSYN assemblies derived from those cases, are delivered into their SN in the presence of additional soluble human αSYN supplied by a rAAV-αSYN expression vector.

### PD, MSA and DLB patient-derived αSYN strains exhibit distinct dopaminergic neurotoxicity

**Fig. 6:**
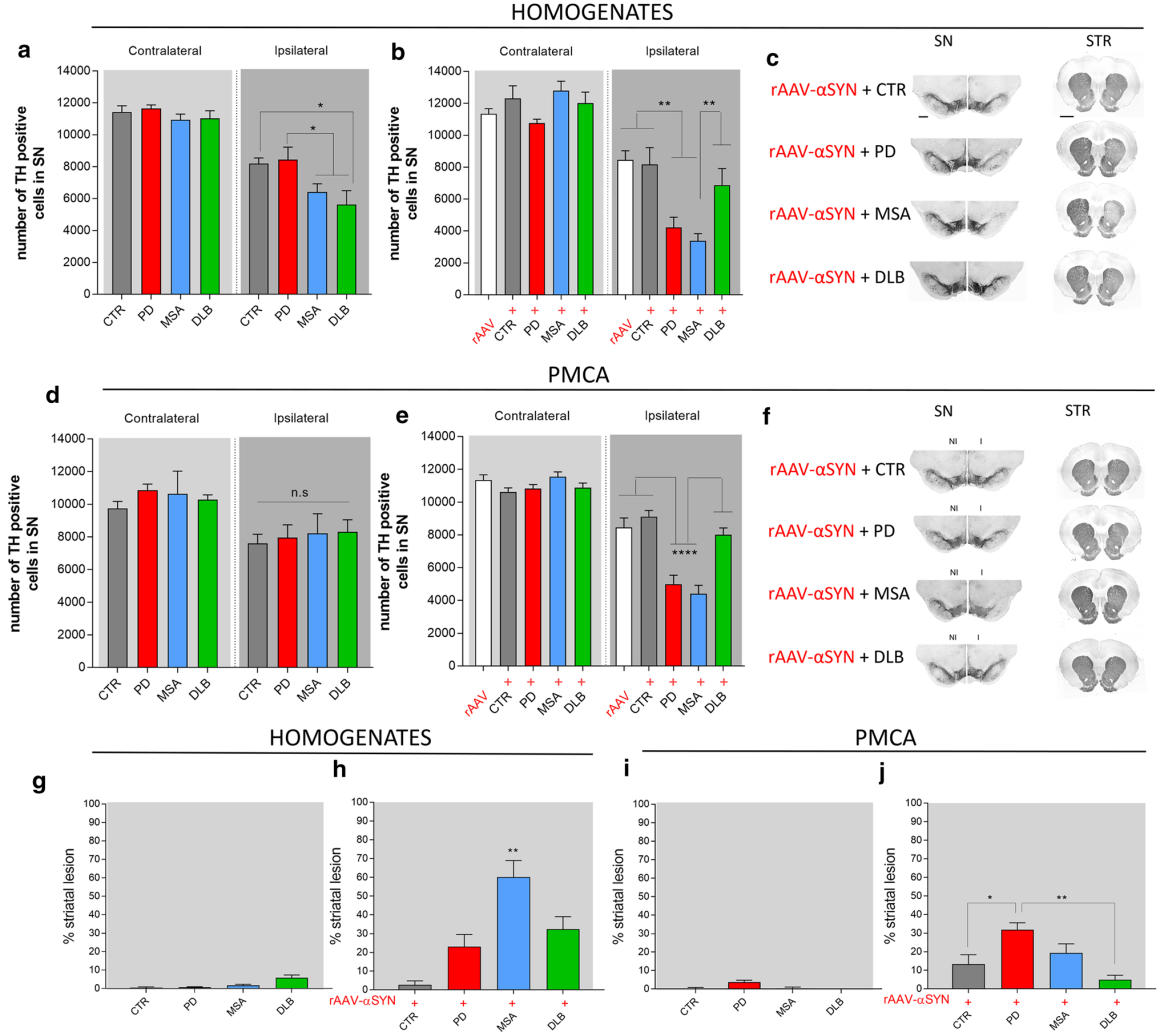
Dopaminergic neurodegeneration after intracerebral inoculation of different patient-derived homogenates and PMCA-amplified αSYN strains. **a**, **b** Stereological quantification of the number of TH^+^ cells in the rat SN 5 months after inoculation of patient-derived homogenates in absence (**a**) or presence (**b**) of rAAV2/7-αSYN overexpression (rAAV). **c** Representative images of TH staining in the SN and striatum five months after co-injection of the patient-derived homogenates and human αSYN overexpression. Scale bar represents 400 µm (SN) and 1000 µm (STR). **d**, **e** Stereological quantification of the number of TH^+^ cells in the rat SN five months after inoculation of PMCA-amplified αSYN in absence (**d**) or presence (**e**) of rAAV2/7-αSYN overexpression. **f** Representative images of TH staining in the SN and striatum five months after co-injection of PMCA-amplified αSYN assemblies and human αSYN expression. Results shown as mean ± SEM (*p < 0.05, **p < 0.01, ****p < 0.0001 for two-way ANOVA with Bonferroni post-hoc analysis, *n* = 4–6 patients per group and *n* = 3 per patient). A total of 12–16 animals per group was included in this study. **g**, **h** TH striatal lesion was assessed 5 months after inoculation of patient-derived homogenates in absence (**g**) or presence (**h**) of rAAV2/7-αSYN overexpression. (**i**, **j**) TH striatal lesion was assessed five months after inoculation of PMCA-amplified αSYN assemblies in absence (**i**) or presence (**j**) of rAAV2/7-αSYN overexpression. Results shown as mean ± SEM (*p < 0.05, **p < 0.01, ****p < 0.0001 for one-way ANOVA with Tukey’s post-hoc analysis, *n* = 4–6 patients per group and *n* = 3 per patient). A total of 12–16 animals per group was included in this study. *I* injected, *NI* non-injected, *n.s.* not significant, *SN* substantia nigra, *STR* striatum

Nigral inoculation of brain homogenates in naive rats led to a decrease in the number of tyrosine hydroxylase (TH) positive neurons with the MSA and DLB groups having the highest proportion of neuronal loss (41 ± 5% and 49 ± 8% respectively) compared to the PD and CTR groups (27 ± 7% and 28 ± 4% respectively) at the final time point of 150 days (Fig. 6a). rAAV-mediated expression of human αSYN resulted in 25 ± 5% nigral dopaminergic cell loss. Upon co-injection with brain homogenates, additional cell loss was observed for the MSA and PD groups (74 ± 3% and 61 ± 6% respectively) but not for the DLB and CTR groups (44 ± 6% and 30 ± 12%; Fig. 6b, c). Nigral inoculation of PMCA-amplified αSYN assemblies in naive rats only induced mild dopaminergic cell loss (ranging from 21–28%) with no differences between the different experimental groups (Fig. 6d). In line with the results obtained with brain homogenates, combining rAAV-αSYN expression with PMCA-amplified MSA and PD assemblies had an additive effect (62 ± 4% and 53 ± 6% respectively) unlike PMCA-amplified DLB and CTR assemblies (26 ± 5% and 14 ± 3%; Fig. 6e, f). Quantifications of striatal dopaminergic nerve terminals further revealed that inoculation of both the brain homogenates or the PMCA-amplified αSYN assemblies in naive rats did not induce a significant striatal lesion at the final time point of 150 days (Fig. 6g, i). However, upon inoculation of brain homogenates in the presence of rAAV-αSYN, significant striatal lesions were induced for all diseases with the most prominent loss for MSA patient brain homogenates (60 ± 8%, Fig. 6h). Similarly, prominent striatal lesions were observed in animals co-injected with rAAV-αSYN and PMCA-amplified assemblies from PD and MSA patients but not from DLB patients (Fig. 6j).

### PD, MSA and DLB patient-derived αSYN strains induce distinct αSYN pathology and spreading

**Fig. 7:**
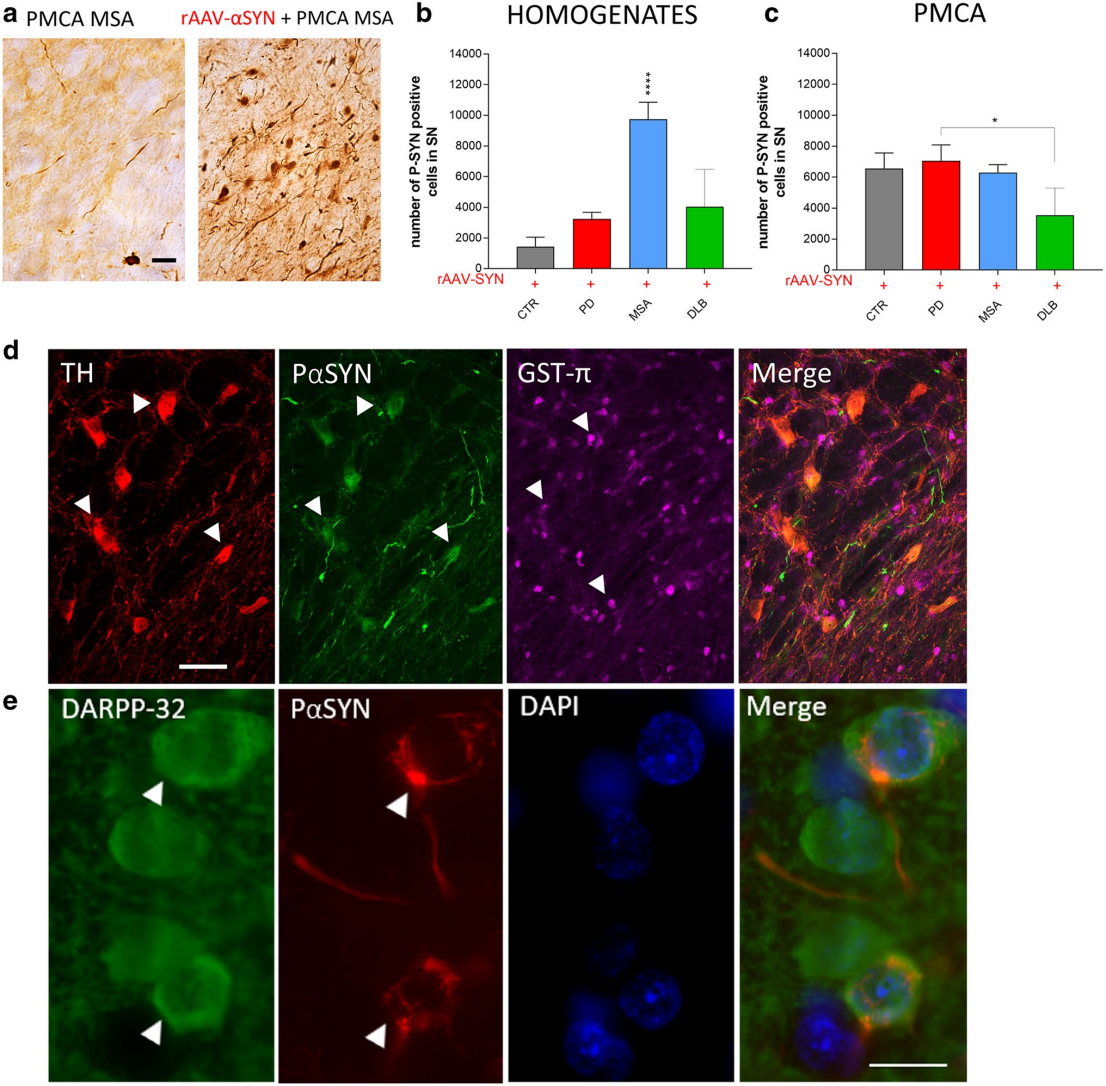
Assessment of αSYN pathology after intracerebral inoculation of different patient-derived homogenates and PMCA-amplified αSYN assemblies in association with rAAV2/7-mediated αSYN expression. **a** Representative images of a P-S129 αSYN staining in the SN 5 months after co-injection of PMCA-amplified MSA strains in absence (left) or presence (right) of rAAV2/7-αSYN overexpression. Scale bar = 50 µm. **b**, **c** Stereological quantification of the number of PαSYN^+^ cells in the SN 5 months after co-injection of patient-derived homogenates (**b**) or PMCA-amplified αSYN assemblies (**c**) with rAAV2/7-αSYN overexpression. Results shown as mean ± SEM (*p < 0.05, ****p < 0.0001 for one-way ANOVA with Tukey’s post-hoc analysis, *n* = 4–6 patients per group and *n* = 3 per patient). A total of 12–16 animals per group was included in this study. **d** Representative images of dopaminergic neurons (TH, in red), αSYN phosphorylated at Ser129 (P-S129 αSYN, in green) and oligodendrocytes (GST-π, in magenta) in the SN 5 months after inoculation of PMCA-amplified MSA strains in association with rAAV2/7-mediated overexpression of αSYN. White arrowheads represent on the one hand neuronal cell bodies (red) co-localizing with P-S129 αSYN staining (green) and on the other hand oligodendroglia cell bodies (magenta) in the absence of co-localization with P-S129 αSYN (green). Scale bar represents 50 µm. **e** Representative image of striatal medium spiny neurons (DARPP-32, in green) and αSYN phosphorylated at Ser129 (P-S129 αSYN, in red) in the striatum 5 months after inoculation of PMCA-amplified MSA strains in association with rAAV2/7-mediated overexpression of αSYN

**Fig. 8:**
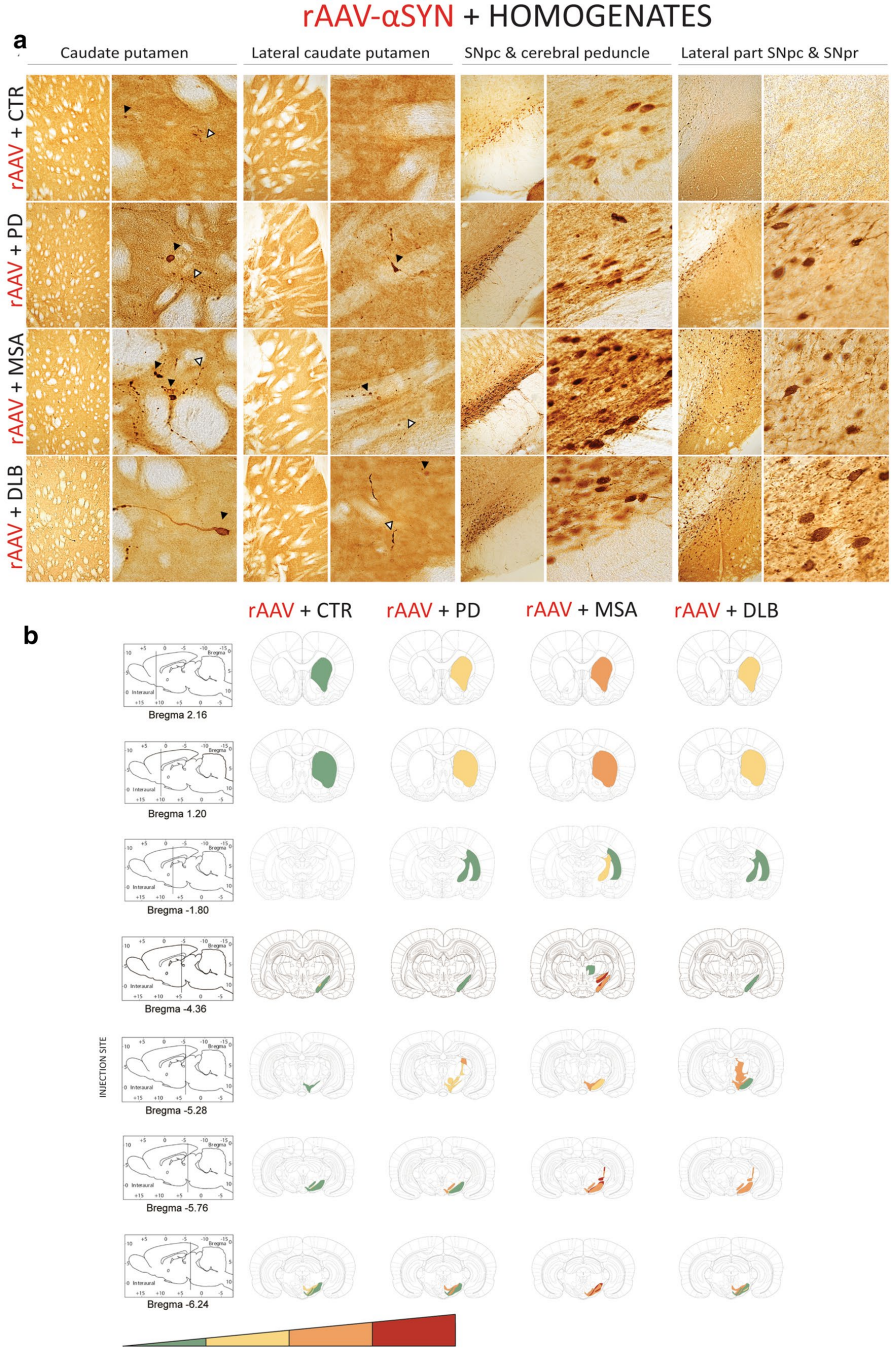
Spreading of αSYN pathology after inoculation of different patient-derived homogenates. **a** Representative photomicrographs of PαSYN pathology in four different brain regions 5 months after co-injection of patient-derived homogenates and rAAV2/7-αSYN overexpression. The selected brain regions are the following: (1) and (2) adjacent sections of caudate putamen (CPu); (3) SNpc and cerebral peduncle (cp); (4) lateral part of the SNpc and SNpr. Black arrows represent PαSYN positive cells and open arrows show PαSYN-positive outgrowths. Scale bars represent 200 µm (left) and 50 µm (right). **b** Heat map for the spreading of αSYN pathology towards different brain regions. The heat map ranges from low (green) tot very high (red). A total of three animals per group was included. The data were presented as heat maps to semi-quantitatively illustrate the distribution of αSYN pathology throughout the brain. Each panel represents a coronal plane (bregma 2.16, 1.20, − 1.80, − 4.36, − 5.28, − 5.76, − 6.24 mm) for each group. The left column presents sagittal views of the corresponding coronal planes

**Fig. 9:**
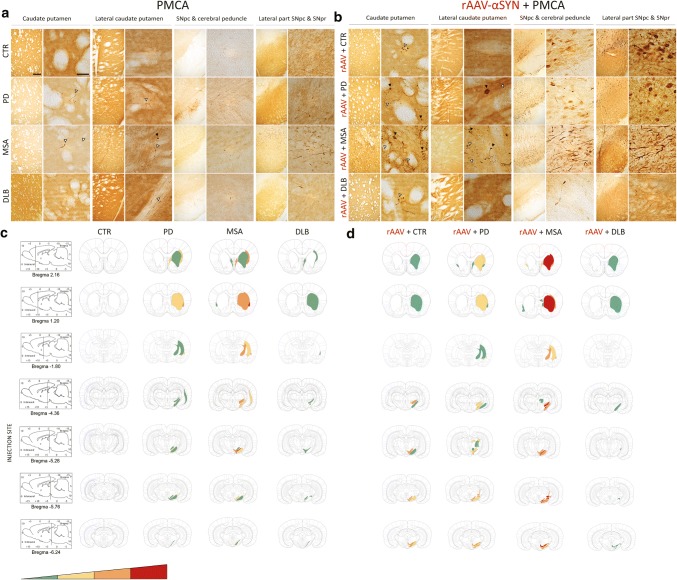
Spreading of αSYN pathology after inoculation of different PMCA-amplified αSYN strains. **a**, **b** Representative photomicrographs of PαSYN pathology in four different brain regions 5 months after inoculation of PMCA-amplified αSYN assemblies without (**a**) or with (**b**) rAAV2/7-αSYN expression. The selected brain regions are the following: (1) and (2) adjacent sections of caudate putamen (CPu); (3) SNpc and cerebral peduncle (cp); (4) lateral part of the SNpc and SNpr. Black arrows represent PαSYN positive cells and open arrows show PαSYN-positive outgrowths. Scale bars represents 200 µm (left) and 50 µm (right). (**c**–**d**) Heat map for the spreading of αSYN pathology towards different brain regions after inoculation of PMCA-amplified αSYN assemblies without (**c**) or with (**d**) rAAV2/7-αSYN expression. The heat map ranges from low (green) tot very high (red). A total of three animals per group was included. The data were presented as heat maps to semi-quantitatively illustrate the distribution of αSYN pathology throughout the brain. Each panel represents a coronal plane (bregma 2.16, 1.20, − 1.80, − 4.36, − 5.28, − 5.76, − 6.24 mm) for each group. The left column presents sagittal views of the corresponding coronal planes

To assess αSYN pathology, we first analyzed the number of PαSYN positive nigral neurons. Injection of PD, MSA and DLB patient brain homogenates or PMCA-amplified assemblies in naive rats resulted in a low number of PαSYN positive dopaminergic neurons (Fig. 7a, left). In contrast, combining rAAV-driven αSYN expression with PD, MSA and DLB patient brain homogenates or PMCA-amplified assemblies led to overall increased numbers of PαSYN positive cells (Fig. 7a, right). Interestingly, the number of nigral PαSYN positive cells was significantly higher in animals of the MSA group when compared to the PD, DLB or CTR groups (Figs. 7b, 8a). This marked PαSYN pathology is in line with the observed severe motor deficits and prominent nigral cell loss (Figs. 5d and 6b). Intriguingly, a significantly lower number of PαSYN positive cells was observed when rAAV-αSYN was co-injected with PMCA-amplified DLB assemblies (Fig. 7c). DLB brain-derived PMCA assemblies appeared to reduce the number of PαSYN cells (Figs. 7c, 9b). The latter results are altogether in agreement with the limited effect of DLB patient-derived PMCA assemblies on motor behavior (Fig. 5f) and nigral and striatal cell loss (Fig. 6e, j). We further examined the presence of PαSYN pathology in other cell types such as astrocytes (data not shown) and oligodendrocytes but we only found PαSYN positive neuronal inclusions in the SN (Fig. 7d) or the striatum (Fig. 7e).

Next, we assessed the distribution pattern of pathological αSYN within the brain following injection of PD, MSA and DLB patient brain homogenates and the corresponding PMCA-amplified assemblies with or without rAAV-driven αSYN expression (Figs. 8, 9). For patient brain homogenates, αSYN phosphorylation patterns in brain regions connected to the injection site were only seen upon co-injection with rAAV-αSYN (Fig. 8a, b). Pathological αSYN deposits were more abundant for all diseases compared to controls but most prominent in animals injected with MSA patient brain homogenates. Both PαSYN positive cells as well as PαSYN positive neurites were observed in the dorsal caudate putamen. A considerable number of PαSYN positive cells and very few PαSYN positive neurites were observed in the SN (Fig. 8a). Interestingly, PMCA-amplified assemblies induced an overall more pronounced PαSYN pathology compared to the patient brain homogenates. The sole injection of PMCA-amplified MSA assemblies induced the most prominent spreading of pathological αSYN deposits in the regions almost similar to those observed with combined injection of rAAV-αSYN and MSA patient brain homogenates (the caudate putamen and the lateral part of the SN, Figs. 7e, 9a, c). However, PαSYN positive neurites were more abundant than PαSYN positive cells. Furthermore, co-injection of rAAV-αSYN and PMCA-amplified MSA assemblies led to a more severe phenotype (Fig. 9b, d). Here, both PαSYN positive cells and PαSYN positive neurites were found in the different brain regions examined. Again, PMCA-amplified DLB assemblies with or without rAAV-αSYN induced very low levels of PαSYN pathology in the striatum, similar to the CTR animals.

**Fig. 10:**
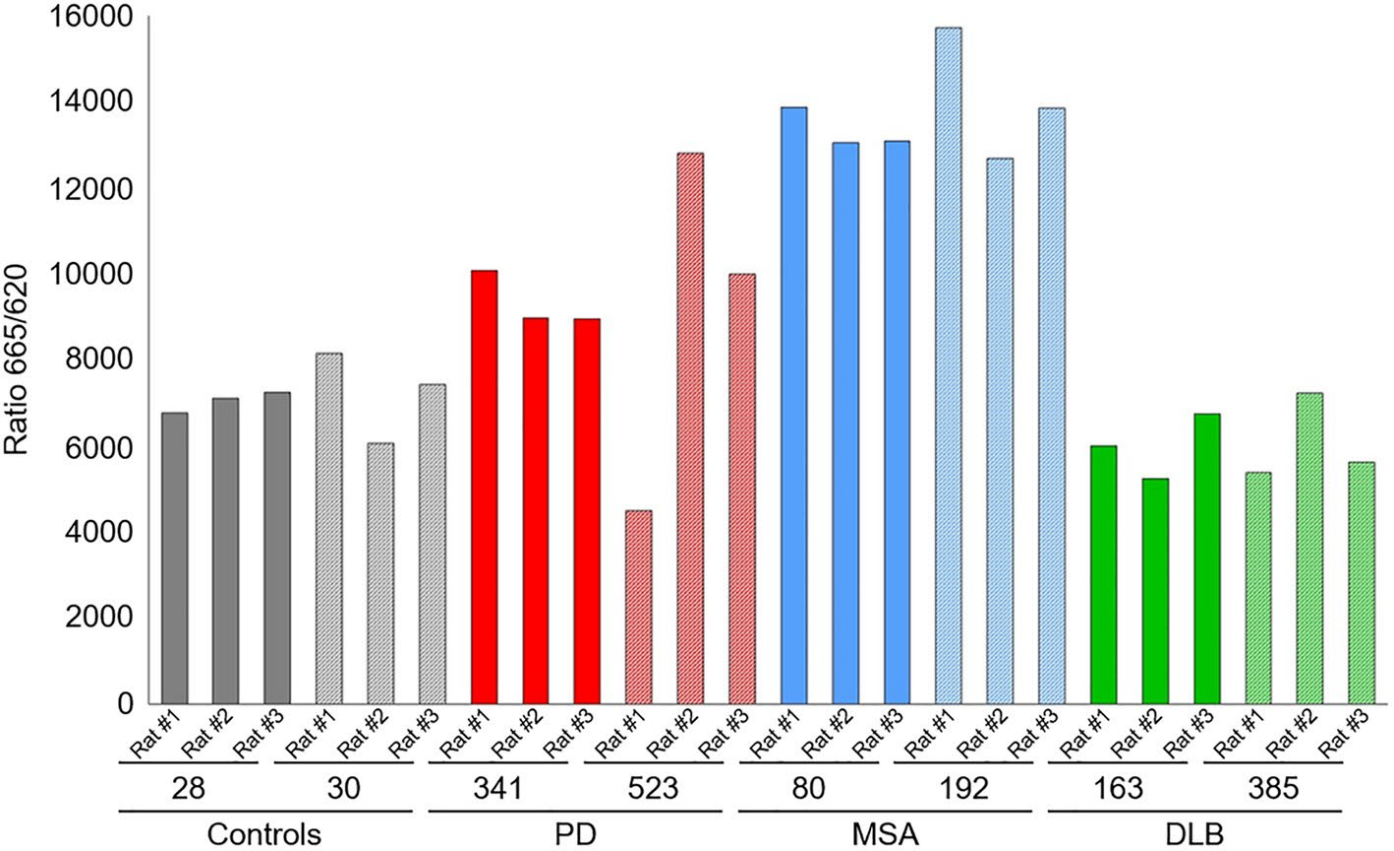
Quantification of aggregated αSYN in rodent brains. The amount of pathogenic, aggregated αSYN in brain homogenates of the rats (2.5%, W:V in PBS) inoculated with the PMCA-amplified αSYN assemblies derived from CTR, PD, MSA and DLB patients (5 µl injected) and rAAV αSYN vector (0.5 µl) was quantified using the Cisbio FRET assay as described in the "Materials and methods” section

The overall amount of aggregated αSYN in the brains of animals who received PD, MSA and DLB patient-derived PMCA-amplified αSYN assemblies together with rAAV-αSYN vector was also quantified using the HTRF Cisbio assay [14]. The amounts of aggregated αSYN in animals injected with PMCA-amplified MSA assemblies were significantly and consistently higher than that of animals of any other group (Fig. 10).

### PD, MSA, and DLB patient-derived αSYN strains trigger a differential immune response

**Fig. 11:**
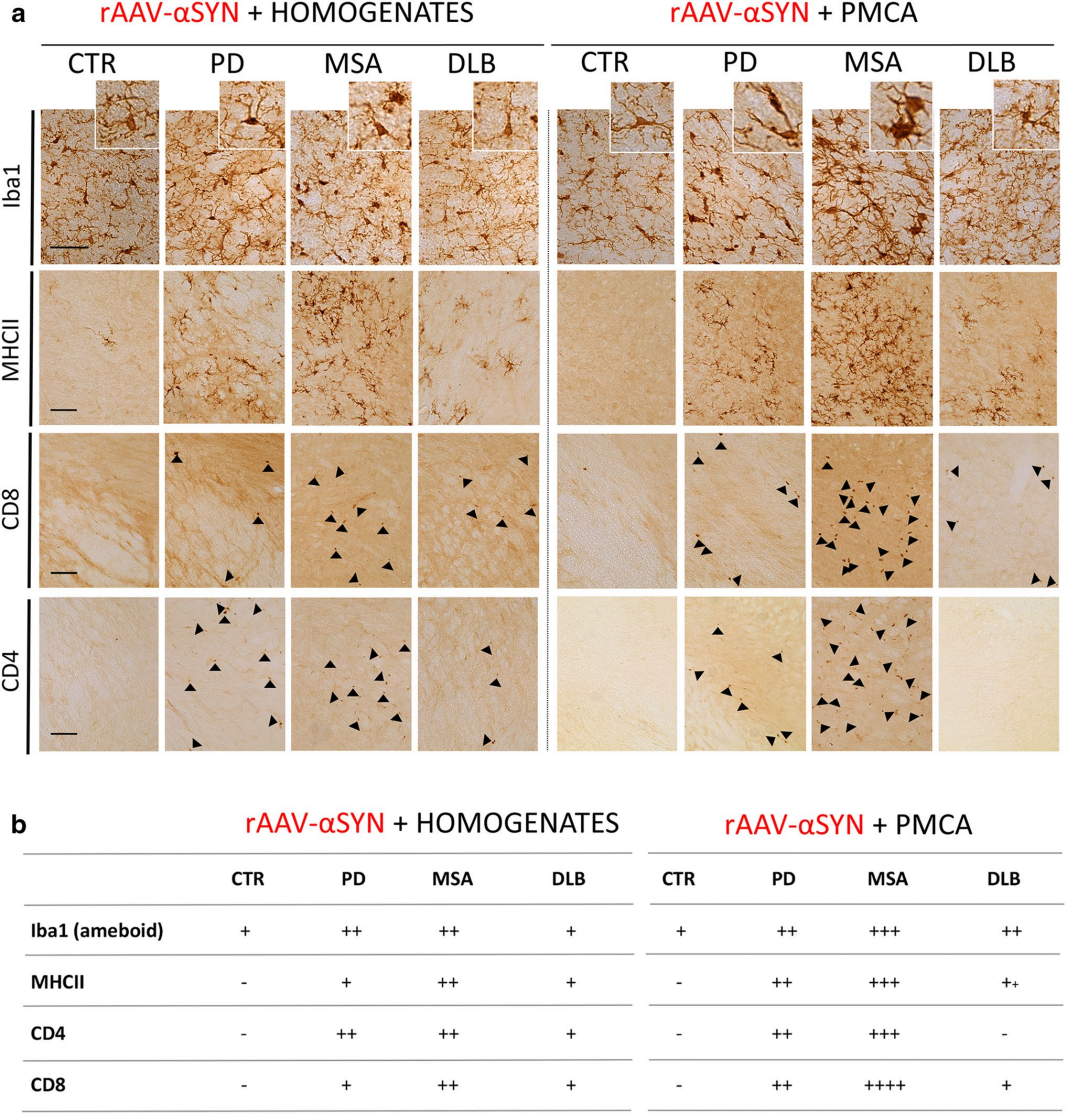
Assessment of immune response after inoculation of different patient-derived homogenates and PMCA-amplified αSYN strains in association with rAAV2/7-mediated αSYN expression. **a** Representative photomicrographs of the immune response stained for Iba1 (microglia), MHCII (reactive microglia), CD4 (T helper cells) and CD8 (cytotoxic T cells) in the SN 5 months after co-injection of different patient-derived homogenates (left) or PMCA-amplified αSYN assemblies (right) with rAAV2/7-αSYN overexpression. Cells immunoreactive for CD4 and CD8 are marked with black arrows. Scale bar Iba1 = 50 µm, MHCII – CD4 = 100 µm. **b** Table with scoring system for the different immune markers Iba1, MHCII, CD4 and CD8 in the rat SN 5 months after inoculation with patient-derived homogenates (left) or PMCA-amplified αSYN assemblies (right) combined with human αSYN expression. The scoring system ranges from not present (−) to abundantly present (++++). A total of three animals per group was included. The data were presented as a table with scoring system to demonstrate the presence of an inflammatory response in the rat SN

To investigate whether distinct αSYN strains may act as antigens altering immune tolerance, provoking neuroinflammation and subsequent deleterious reactions, we investigated the presence of different immune-related cells in the brain. In naive animals, injection of different brain homogenates or PMCA-amplified αSYN strains did not induce a detectable Iba1 positive microglial response at the final time point of 150 days (data not shown). However, in the presence of human αSYN, we detected a pronounced additive immune response. Remarkably, the overall immune response to PMCA-amplified αSYN assemblies from PD, MSA and DLB patients was more significant compared to the corresponding brain homogenates. When comparing diseases, MSA-derived material induced the strongest immune response, followed by PD and DLB (Fig. 11). More specifically, co-injection of PD or MSA brain homogenates or PMCA-amplified αSYN strains with rAAV-αSYN resulted in a significantly higher number of Iba1  positive cells and the presence of large phagocytic reactive microglia (Fig. 11a, b). Major histocompatibility complex class II  (MHC II) expression was triggered by injection of PD, MSA and DLB material, with the strongest expression for samples originating from MSA patients (Fig. 11a, b). Since microglial upregulation of MHC II is suggestive of an adaptive immune response, we further examined the affected areas for CD4 (helper) and CD8 (cytotoxic) T cell infiltration. An increase in the number of CD4 and CD8 positive T cells paralleled MHCII expression (Fig. 11a, b).

## Discussion

The discovery that αSYN assembles into structurally distinct fibrillar assemblies has led to the hypothesis that those assemblies, also referred to as ‘strains’, could account for the different clinicopathological traits within synucleinopathies [9, 36]. In a previous study, we brought evidence for a structural-pathological relationship for αSYN strains in synucleinopathies, by showing that two recombinant αSYN fibrillar polymorphs, which possessed different structural and seeding properties, trigger distinct histopathological and behavioral phenotypes in vivo [36]. Here, we present evidence that distinct αSYN strains exist in well-stratified cohorts of PD, MSA and DLB patients. We used the seeding propensity of aggregated αSYN in diseased human brain homogenates in an ex vivo assay inspired by the PMCA method [17] to amplify and generate pure and homogenous assemblies of αSYN that are disease-specific. Our observations reveal unique seeding features of brain homogenates from patients who developed PD, DLB or MSA in agreement with observations made using a different amplification method [11]. Besides exhibiting different shapes upon TEM analysis, the amplified fibrillar assemblies showed distinct fingerprints, characteristic of PD, MSA and DLB. Indeed, the limited proteolysis patterns of PD and MSA patient-derived fibrils shared significant similarities and differed from those derived from DLB patients. The proteolysis pattern of PD and MSA patient-derived fibrils resembled that of the fibrillar polymorph ‘Ribbons’ while the pattern of DLB patient-derived fibrils was similar to the fibrillar polymorph ‘Fibrils’. In addition, PD and MSA patient-derived fibrils are overall recognized to a lesser extent than DLB patient-derived fibrils by the aggregated αSYN-specific antibody FILA4. Altogether, these findings strongly suggest that pathogenic αSYN in PD, DLB and MSA has distinct structural traits that are characteristic of each synucleinopathy and can be reproduced through the amplification assay we implemented. Our data further indicate that TEM analysis, proteolytic and conformational antibodies profiling provide complementary information, critical to distinguish one synucleinopathy from another. Our results are in partial agreement with a recent study demonstrating similarities between PMCA-amplified fibrils derived from PD and MSA patient brains, although significant structural diversity between individual patients was also reported, while we found mostly similarities between patients from the same disease [52].

In a cellular seeding assay and in primary neurons, all patient-derived αSYN assemblies induced aggregation and phosphorylation of αSYN, which demonstrates the seeding capacity of all amplified fibrillar assemblies. This is in line with previously published data for recombinant αSYN seeds [29, 34, 58]. Somewhat surprisingly, and in contrast with previous published data [44], the brain homogenates did not result in significant inclusion formation under our experimental conditions. This is most probably due to the amount of seeds in the diluted brain homogenates. In addition, in spite of some variability between individual patients, we observed disease-specific differences in the number and shape of the aggregates induced by PMCA-amplified strains. αSYN strains derived from DLB patients induced a higher number of inclusions with a longer shape compared to PD and MSA αSYN strains in the human H4 neuroglioma cells seeding assay. In primary neurons, DLB-derived αSYN strains induced diffuse PαSYN deposits within the soma surrounding the nucleus, while PD and MSA strains led to mainly neuritic linear or punctate PαSYN positive inclusions. These data suggest either differential seeding, take-up or clearing propensities for distinct patient-derived αSYN strains.

Next, we assessed the properties of patient-derived brain homogenates as well as PMCA-amplified αSYN strains after intracerebral inoculation in the rat SN. Overall, there were considerable similarities but also some differences for the different phenotypic read-outs between brain homogenates and fibrils obtained by PMCA. Of course, one needs to take into consideration that total brain homogenates are heterogeneous in nature and contain beside aggregated αSYN a mixture of proteins and probably inflammatory components, whereas PMCA-derived assemblies represent pure αSYN protein. In this regard, we propose that phenotypic traits induced by PMCA-derived assemblies may be attributed to strain-specific properties, while phenotypes induced by patient-derived homogenates may be explained by a combination of differences in strain and/or disease environment. When comparing to our previous findings for recombinant Fibrils and Ribbons in vivo, the MSA- and PD-derived strains partially resemble Ribbons with regard to the induction of αSYN pathology, while in terms of potency to induce motor deficits, dopaminergic neurodegeneration and neuroinflammation, they appear more similar to Fibrils [36]. This suggests that the patient-derived assemblies represent distinct strains with similarities but also differences compared to the recombinant strains.

The induction of αSYN pathology throughout the brain after inoculating MSA or DLB-derived brain homogenates in the parietal lobe, the striatum or the SN of transgenic mice has been reported previously [8, 30, 44, 63]. Progressive nigrostriatal neurodegeneration upon intranigral inoculation of PD-derived αSYN-enriched brain fractions in both mice and monkeys has also been described [45]. More recent studies using sarkosyl-insoluble αSYN fractions from brains of patients with MSA or Lewy body disease (grouping Alzheimer’s disease, DLB and Parkinson’s disease Dementia  (PDD) together) have suggested that both the αSYN seed and its environmental context determine how αSYN strains might originate [37].

Here, to the best of our knowledge, we compared for the first time the structural and pathogenic characteristics of brain homogenates in parallel with pure PMCA-amplified αSYN strains from well-stratified synucleinopathy patients that were clinically and neuropathologically examined. MSA patient brain homogenates as well as MSA PMCA-derived strains—especially when combined with viral vector-mediated human αSYN expression—appear to be most potent in inducing motor deficits, nigrostriatal neurodegeneration, αSYN pathology and spreading, which reflects the aggressive nature of MSA. The more pronounced phenotype observed in the presence of soluble human αSYN supplied by rAAV vectors, as previously described [36, 56], is in line with the accelerated proliferation observed in transgenic M83 mice compared to wild type animals [26]. Of note, we were unable to detect clear oligodendroglial inclusions in any experimental condition, which is in agreement with previous studies [37, 44, 63]. Interestingly, PD patient-derived αSYN strain resembled the strain originating from MSA patients. The two strains had similar shapes and fingerprint. Both strains affected similarly dopaminergic neuron survival but the PD-derived αSYN strain appears to template αSYN aggregation in a less efficient manner, with as a consequence a less pronounced disease phenotype. The latter may be the consequence of either a slower growth rate, a lower resistance to clearance or to the ability of this strain to propagate from one cell to another. Our finding that DLB brain homogenates and their corresponding PMCA-amplified αSYN assemblies, appear to be much less pathogenic, yielding a milder disease phenotype was somewhat unexpected. Until now, only a limited number of studies have investigated DLB-derived αSYN strains. Amplification of the frontal cortex and the SN by RT-QuIC revealed stronger seeding activity for DLB compared to PD, but this study did not include in vivo characterization [11]. Moreover, unlike PMCA, RT-QuIC amplification reactions are performed in the presence of the dye thioflavin T. This ligand could facilitate, through its differential binding to distinct fibrillar αSYN fibrillar assemblies [9], the formation of strains that differ from those obtained by PMCA. In addition, the brain regions examined might also be relevant, since regional variation was described for different synucleinopathies [50]. The intrinsic structure of pathogenic αSYN may depend on the local conditions of the brain region where they formed. Once formed, those aggregates propagate in a prion-like manner to other affected regions. By doing so, they imprint their intrinsic structure to endogenous monomeric αSYN. Thus, it might be interesting in the future to compare structurally seeds from different brain regions for each synucleinopathy. Finally, the differential behavior of αSYN strains was demonstrated very recently for recombinant, MSA and DLB brain-derived αSYN strains in M83 A53T-αSYN transgenic mice [26]. In agreement with our study, MSA-derived assemblies induced the most severe phenotype, while DLB assemblies did not propagate efficiently.

The recent observations that aggregated αSYN templates the aggregation of the monomeric form of the protein and spread throughout the nervous system, suggest that αSYN aggregation drives synucleinopathy progression. However, the role of inflammatory processes in this complex scenario is poorly understood. Extracellular propagation of distinct αSYN strains could alter the immune tolerance and subsequent reaction, leading to neurotoxicity through the release of pro-inflammatory cytokines. Previously, we observed brain infiltration of T cells in our rAAV2/7-αSYN rat model, suggesting a link between adaptive immunity and synucleinopathy [38]. In this study, we demonstrate a strong Iba1 and MHCII response (antigen presentation) as well as CD4 and CD8 T cell infiltration upon inoculation with PD and MSA patient-derived strains. The differential immune response toward distinct αSYN strains shows that the synucleinopathy strains represent unique antigens, which might determine disease outcome. In line with our results, well-defined αSYN peptides and structurally distinct αSYN assemblies drive helper and cytotoxic T cell responses and activate monocytes in PD patients [22, 54], thus corroborating the GWAS link to specific major histocompatibility complex alleles [2].

With this work, we characterized distinct human αSYN strains in vivo and provide new evidence that underline their relevance in synucleinopathies. We demonstrate that a specific signature can be attributed to PD, MSA and DLB-derived strains that differs from previously described recombinant strains (Table 2). Future studies investigating how distinct patient-derived αSYN strains behave when specifically targeting different brain regions (e.g. MSA strains in the basal ganglia (striatum) and the cerebellum or DLB strains in the neocortex) will further unravel this complex question. The development of highly sensitive techniques with the aim of detecting pathological αSYN strains might contribute to earlier and specific diagnosis of synucleinopathies. Therapeutic strategies specifically targeting disease-specific strains or their unique interactome might open new avenues aimed at slowing or stopping disease progression.

**Table 2:** The table summarizes the data obtained after inoculation with the different patient-derived homogenates (left) or PMCA-amplified αSYN assemblies (right) in association with human αSYN expression

	rAAV-αSYN + HOMOGENATES				rAAV-αSYN + PMCA			
	CTR	PD	MSA	DLB	CTR	PD	MSA	DLB
Motor deficits			+					−
DA degeneration SN		+	+			+	+	
Stiatal nerve loss			+					−
αSYN pathology SN			+					−
αSYN spreading			+				++	−
Immune response			+				++	−

The following parameters are included: motor deficits, dopaminergic neuronal cell loss in the SN, striatal nerve loss, αSYN pathology in the SN, spreading of αSYN pathology and immune response. The scoring system ranges from not/mildly present (−) to strongly present (++)

α*SYN* α-synuclein, *DA* dopaminergic, *rAAV-αSYN * recombinant adeno-associated viral vector expressing αSYN, *SN* substantia nigra
